# Single-nuclei isoform RNA sequencing unlocks barcoded exon connectivity in frozen brain tissue

**DOI:** 10.1038/s41587-022-01231-3

**Published:** 2022-03-07

**Authors:** Simon A. Hardwick, Wen Hu, Anoushka Joglekar, Li Fan, Paul G. Collier, Careen Foord, Jennifer Balacco, Samantha Lanjewar, Maureen McGuirk Sampson, Frank Koopmans, Andrey D. Prjibelski, Alla Mikheenko, Natan Belchikov, Julien Jarroux, Anne Bergstrom Lucas, Miklós Palkovits, Wenjie Luo, Teresa A. Milner, Lishomwa C. Ndhlovu, August B. Smit, John Q. Trojanowski, Virginia M. Y. Lee, Olivier Fedrigo, Steven A. Sloan, Dóra Tombácz, M. Elizabeth Ross, Erich Jarvis, Zsolt Boldogkői, Li Gan, Hagen U. Tilgner

**Affiliations:** 1 Feil Family Brain and Mind Research Institute, Weill Cornell Medicine, New York, NY USA; 2 Center for Neurogenetics, Weill Cornell Medicine, New York, NY USA; 3 Helen and Robert Appel Alzheimer’s Disease Research Institute, Weill Cornell Medicine, New York, NY USA; 4 The Rockefeller University, New York, NY USA; 5 Department of Human Genetics, Emory University School of Medicine, Atlanta, GA USA; 6 Department of Molecular and Cellular Neurobiology, Center for Neurogenomics and Cognitive Research, Amsterdam Neuroscience, VU University, Amsterdam, The Netherlands; 7 Center for Algorithmic Biotechnology, Institute of Translational Biomedicine, St. Petersburg State University, St. Petersburg, Russia; 8 Physiology, Biophysics & Systems Biology Program, Weill Cornell Medicine, New York, NY USA; 9 Agilent Technologies, Santa Clara, CA USA; 10 Human Brain Tissue Bank, Semmelweis University, Budapest, Hungary; 11 Department of Medicine, Division of Infectious Diseases, Weill Cornell Medicine, New York, NY USA; 12 Center for Neurodegenerative Disease Research, University of Pennsylvania School of Medicine, Philadelphia, PA USA; 13 Department of Medical Biology, Albert Szent-Györgyi Medical School, University of Szeged, Szeged, Hungary

**Keywords:** Transcriptomics, Molecular neuroscience, Autism spectrum disorders, Medical genomics

## Abstract

Single-nuclei RNA sequencing characterizes cell types at the gene level. However, compared to single-cell approaches, many single-nuclei cDNAs are purely intronic, lack barcodes and hinder the study of isoforms. Here we present single-nuclei isoform RNA sequencing (SnISOr-Seq). Using microfluidics, PCR-based artifact removal, target enrichment and long-read sequencing, SnISOr-Seq increased barcoded, exon-spanning long reads 7.5-fold compared to naive long-read single-nuclei sequencing. We applied SnISOr-Seq to adult human frontal cortex and found that exons associated with autism exhibit coordinated and highly cell-type-specific inclusion. We found two distinct combination patterns: those distinguishing neural cell types, enriched in TSS-exon, exon-polyadenylation-site and non-adjacent exon pairs, and those with multiple configurations within one cell type, enriched in adjacent exon pairs. Finally, we observed that human-specific exons are almost as tightly coordinated as conserved exons, implying that coordination can be rapidly established during evolution. SnISOr-Seq enables cell-type-specific long-read isoform analysis in human brain and in any frozen or hard-to-dissociate sample.

## Abstract (web summary)

Complete RNA isoforms are captured by a new single-nuclei sequencing method.

## Main

Concurrent with the development of single-cell RNA sequencing^1–3^, long-read approaches enabled complete isoform analysis^4–8^. More recently, long reads empowered the analysis of a few^9,10^, and then thousands of, single cells^11,12^ using high-throughput single-cell approaches, including 10x Genomics.

**Fig. 1:**
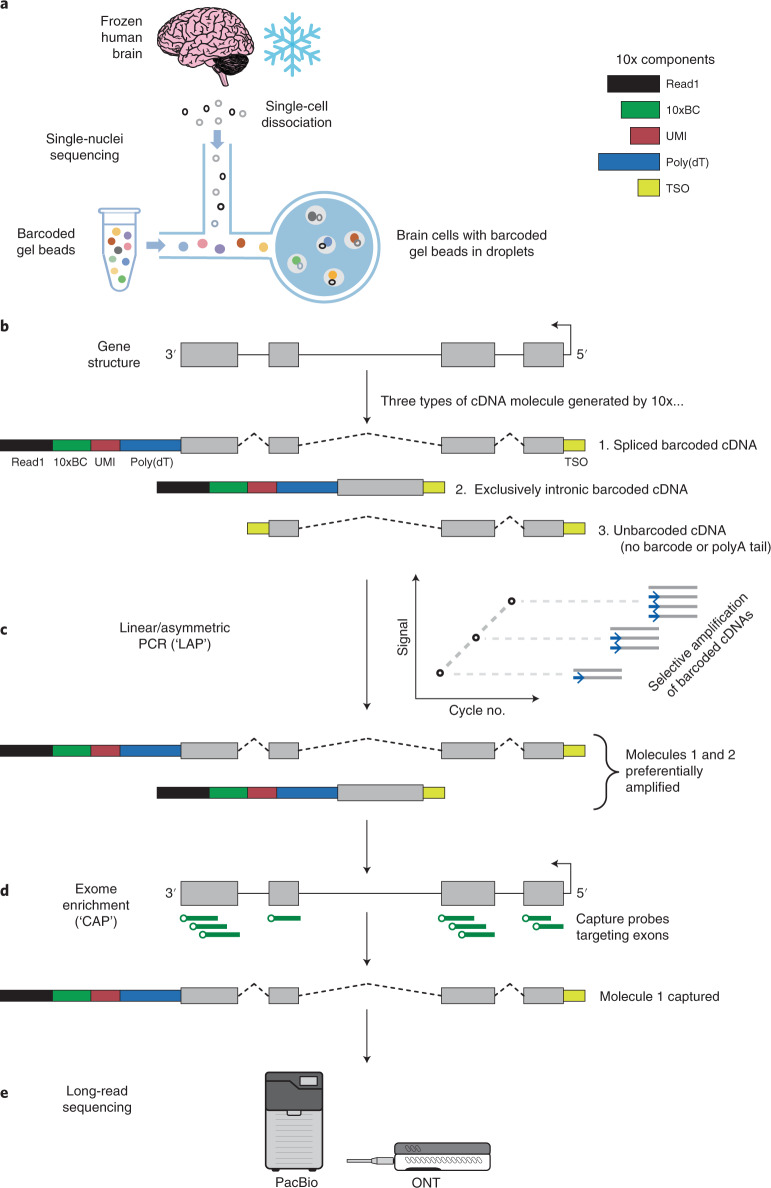
Overview of the SnISOr-Seq approach. **a**, Barcoded cDNA library of nuclei isolated from frozen human brain tissue. **b**, Three main types of molecules generated: spliced barcoded (known and novel isoforms), unspliced barcoded (exclusively intronic nucleotides) and incomplete cDNA without a cellular barcode. **c**, Linear/asymmetric PCR (‘LAP’) is used to selectively amplify barcoded cDNA. **d**, Probe-based exome capture (‘CAP’) step is applied to filter out purely intronic cDNA molecules. **e**, Molecules are sequenced on a long-read sequencer (PacBio and ONT).

Single-nuclei methods^13–15^ are widely used for many applications and especially for frozen tissues, including human brain (Fig. 1a). Single-nuclei datasets contain many partially or fully unspliced RNAs, leading to many reads derived from purely intronic regions. These reads are reverse-transcribed from genomically encoded polyadenylation (polyA)-rich regions or through artifacts and are usable for gene count and ‘RNA velocity’ analyses^16–18^. However, such intronic reads cannot inform on complete isoforms. Another problem for long-read sequencing of 10x Genomics single-nuclei and single-cell libraries are molecules lacking polyA tails, barcodes and Illumina adaptors (Fig. 1b). Such cDNAs are biased against in Illumina library preparation and sequencing but sequenced on Pacific Biosciences (PacBio)^19^ and Oxford Nanopore Technologies (ONT) platforms, which do not require Illumina adaptors. Here we present single-nuclei isoform RNA sequencing (SnISOr-Seq), which overcomes both above problems. In brief, we employ linear/asymmetric PCR, amplifying full-length cDNAs from the 10x Genomics partial-read1, near which polyA tails and barcodes reside. This step enriches for polyA-tail-containing and barcode-containing molecules (Fig. 1c). Second, we use enrichment probes to select cDNA molecules overlapping exons, thereby removing purely intronic molecules (Fig. 1d). We collectively refer to these linear/asymmetric PCR and capture steps as ‘LAP-CAP’. We then long-read sequence these post-LAP-CAP molecules (Fig. 1e). SnISOr-Seq can detect multiple splicing events in barcoded long reads, which might originate from genuine polyA sites as well as internal polyA-rich regions.

Using SnISOr-Seq, we investigate how distinct transcript elements—alternative transcription start sites (TSSs), exons and polyA sites—are combined into full-length isoforms in the human brain and determine the cell-type-specific basis of coordination events. We and others have previously investigated the coordination of exon pairs, TSSs and polyA sites genome-wide^7,20,21^ or specifically for neurexins^22,23^. Mechanisms underlying exon–exon coordination and the influence of promoters on splicing are established^24,25^ for individual genes. Splicing can also influence TSS choice^26^, and interactions between splicing and 3′-end cleavage have also been described^27^. Likewise, the order of intron removal from the pre-mRNA has been tackled in yeast^28^. However, how transcript element combinations specify cell types in the human brain remains unknown, limiting understanding of brain function. Similarly to the use of single alternative exons, the coordination status of transcript elements observed in bulk can have origins from coordination in specific cell types or also from distinct isoforms in distinct cell types. We found that TSS–exon and exon–polyA site coordination follows a similar model to the coordination of distant alternative exons, whereas adjacent alternative exons follow a different model for cell type usage. Alternative splicing mis-regulation in disease is established^22,29,30^; however, whether these exons are independently affected or hijacked in coordinated units is unknown. Using SnISOr-Seq’s capacity for cell-type-specific long-read sequencing, we found that exons associated with distinct diseases exhibit distinct behavior in terms of (1) inclusion variability across cell types and (2) coordination. Despite common cortical roots, autism spectrum disorder (ASD)-associated exons show markedly different behavior than schizophrenia-associated and amyotrophic lateral sclerosis (ALS)-associated exons, with the caveat that distinct methods defined the exons associated to each disease.

## Results

### Removing single-cell artifacts and unspliced RNAs

**Fig. 2:**
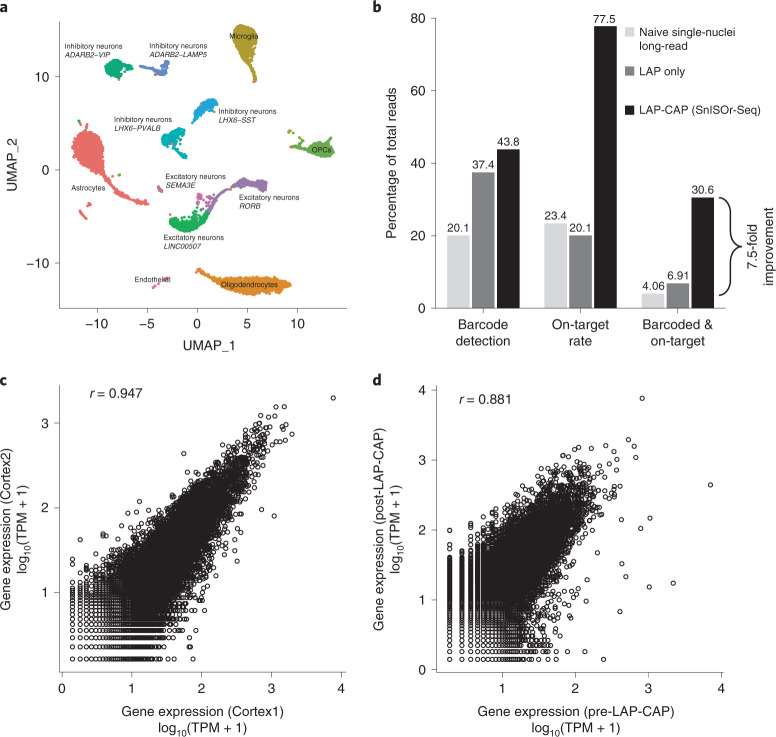
Cell type clustering and enrichment efficiency. **a**, UMAP plot of the Cortex1 sample with each point representing a single nucleus and colors indicating cell type. **b**, Bar plot showing the barcode detection rate, on-target rate and fraction of reads that are usable (that is, barcoded and on-target). Color of bars indicates experimental approach: naive single-nuclei long read (light gray) as a control; LAP (dark gray); SnISOr-Seq (black). **c**, Scatter plot of the correlation in PacBio long-read gene expression (log_10_ TPM + 1) between Cortex1 and Cortex2. Pearson correlation (*r*) is indicated. **d**, Scatter plot of the correlation in PacBio long-read gene expression for Cortex1 before and after LAP-CAP. TPM, transcripts per million.

We first performed single-nuclei 3′-end sequencing of frontal cortex tissue from two healthy donors aged 68 and 61 years old from the Penn Brain Bank (henceforth referred to as ‘Cortex1’ and ‘Cortex2’; Methods). Employing standard protocols for single-cell analysis^31,32^, we defined 12 clusters representing all major cortical cell types, including neurons, astrocytes, oligodendrocytes, microglia and vascular cells. Among neurons, we observed multiple inhibitory neuron types, including *SST*^+^, *LAMP5*^+^ and *PVALB*^*+*^ interneurons, and layer-specific excitatory neuron types: *RORB*^+^*, SEMA3E*^+^ and *LINC00507*^*+*^ (Fig. 2a and Supplementary Fig. 1a–r). We sequenced 8,376 unique molecular identifiers (UMIs) per cell of Cortex1, with excitatory neurons (subtype *RORB* and *SEMA3E*) showing the highest UMI counts per nucleus and astrocytes and oligodendrocytes the lowest (Supplementary Fig. 2a). These UMI statistics were mirrored by similar gene-per-nucleus trends (Supplementary Fig. 2b). Both Cortex1 and Cortex2 showed high percentages of reads attributed to nuclei and low antisense mappings (Supplementary Fig. 2c). We then used 500 ng of full-length cDNA and performed linear/asymmetric PCR and Agilent exome enrichments (LAP-CAP; Methods), followed by exponential/symmetric PCR. The resulting cDNAs were sequenced on eight (Cortex1) and seven (Cortex2) PacBio SMRT cells and three (Cortex1) and two (Cortex2) ONT PromethION flow cells. This yielded ~290 × 10^6^ long reads with average lengths of 0.9–1.2 kb across technologies and samples (Supplementary Table 1). As a negative control, we sequenced one SMRT cell per sample before LAP-CAP and one after LAP. We detected barcodes in long reads as recently published^12,33^. The barcoded read fraction increased strongly from naive single-nuclei long-read sequencing to LAP-CAP (Fig. 2b). Likewise, on-target reads were markedly more frequent in LAP-CAP (Fig. 2b). We observed strong correlation in gene expression between Cortex1 and Cortex2 (*r* = 0.947; Fig. 2c), demonstrating SnISOr-Seq’s replicability. When using all mapped reads (barcoded and unbarcoded), the correlation observed between Cortex1 before and after LAP-CAP was relatively strong (*r* = 0.881; Fig. 2d). However, SnISOr-Seq yielded a ~7.5-fold-higher fraction of ‘usable’ reads (that is, reads that were mapped, barcoded and on-target) compared to naive long-read single-nuclei sequencing (30.6% versus 4.06%; Fig. 2b).

We found that, despite being deployed in a considerably more complex environment (frozen tissue, nuclei and large postmortem intervals), SnISOr-Seq was almost on par with ScISOr-Seq in fresh cells for transcript coverage bias, read length and exon count. Read length differences accounted for much, but not all, of the observed coverage differences between short and long reads (Methods and Supplementary Fig. 3a–c). We consider a read 5′ and/or 3′ complete if the start and/or end overlap a 50-bp window of published Cap Analysis of Gene Expression (CAGE) and polyA peaks, respectively (Supplementary Fig. 3d and Methods). We found that SnISOr-Seq provides fewer complete molecules, probably due to intron retention and the fragmented nature of nuclear RNA from postmortem tissue. Especially on the 3′ end, large introns are detrimental to producing full-length molecules (Supplementary Fig. 3e,f). Consequently, SnISOr-Seq covers ~57.1% of the expected exons per transcript in each read, whereas ScISOr-Seq yields close to all expected exons (Methods and Supplementary Fig. 3g,h). Subsampling simulations showed that genes and pairs of isoform features all approached saturation at full sequencing depth (Supplementary Fig. 3i). Similarly to recent bulk PacBio RNA sequencing of human cortex^34^, detected genes plateaued at ~12,000. For initiating reverse transcription, simulations suggest that poly(dT) priming captures entire polyadenylated molecules. However, RNA fragments lacking a polyA tail might be missed by poly(dT) primers, whereas some of their sequence might be captured by random hexamers (Methods and Supplementary Fig. 4a). At a sequencing depth of ~1.1 million long reads, LAP-CAP sample had one UMI per 1.06 barcoded reads compared to one UMI per 1.001 for the pre-LAP-CAP sample (Supplementary Fig. 4b). However, for 20 × 10^6^ long reads, the LAP-CAP sample yielded one UMI per 1.46 reads (Supplementary Fig. 4c,d). SnISOr-Seq in human nuclei even outperformed ScISOr-Seq in fresh mouse samples in usable reads, and both methods were on par for exons per spliced read (Supplementary Fig. 4e,f). Lastly, SnISOr-Seq was clearly advantageous in recovering fully spliced reads as compared to unspliced and partially spliced reads (Supplementary Fig. 4g,h), although only 41% of 5′ read ends and 52% of 3′ read ends corresponded to CAGE and polyA peaks, respectively (Supplementary Fig. 3e,f).

The ONT datasets had 515 and 384 median reads per nucleus for Cortex1 and Cortex2, respectively (Supplementary Fig. 5a,b). The four major cell types (astrocytes, oligodendrocytes, excitatory neurons and inhibitory neurons) represented 77.9% (Cortex1) and 82.6% (Cortex2) of nuclei (Supplementary Fig. 5c,d), and excitatory neurons consistently had the most reads, UMIs and genes per nucleus (Supplementary Fig. 5e–j). Of note, excitatory neurons had higher counts in Cortex2, mostly at the expense of oligodendrocytes and astrocytes (Supplementary Fig. 5). The ONT LAP-CAP data were sequenced to greater depth than the PacBio libraries. However, both datasets highly correlated for reads per gene and identified splice sites and exon inclusion levels (Supplementary Fig. 6a–d).

### Single-exon patterns reveal variable inclusion across cell types, including for ASD-associated exons

**Fig. 3:**
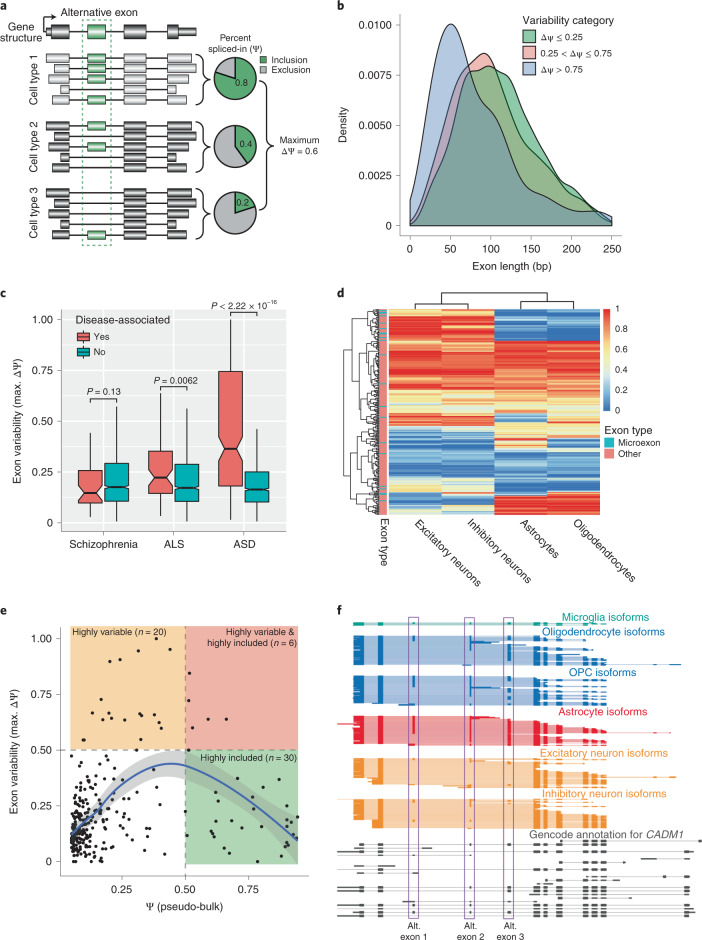
Alternative usage of single exons. **a**, Schematic illustrating percent spliced-in (*Ψ*) calculation for an alternative exon (green). The exon shows different levels of inclusion across three cell types, with variability defined as max*Ψ* − min*Ψ*. **b**, Density plot of the exon variability across the four major cell types and exon length on the *x* axis. Colors indicate the discrete categories of variability. **c**, Box plots of the exon variability for alternative disease-associated exons (red) compared to alternative exons with no known association with that disease (green). *P* values obtained from a two-sided Wilcoxon rank-sum test. Investigated diseases are represented on the *x* axis (*n* = 46; 1,580; 69; 1,557; 227; and 1,399 exons). **d**, Heat map of the exon inclusion level for ASD-associated exons where each row is an exon and each column is a cell type. Annotation of exon classification as a microexon (≤27 bp; green) and other exons (>27 bp; pink) on the left. Color scale of the heat map indicates *Ψ* value. **e**, Scatter plot of the *Ψ* of pseudo-bulk (that is, across all nuclei) on the *x* axis and exon variability across cell types on the *y* axis. Points indicate novel exons that had ≥10 reads in ≥2 cell types. Regression curve with 95% confidence interval obtained using the loess fit. Boundaries of low and high variability and inclusion defined at 0.5 on both axes. **f**, Full-length transcript expression by cell type for the *CADM1* gene. Each horizontal line indicates one transcript, colored by cell type; clustered blocks denote exons. Black denotes annotated GENCODE transcripts. Purple boxes highlight three alternative exons: AE1–AE3. For box plots: center line, median; box limits, upper and lower quartiles; and whiskers, 1.5× interquartile range.

Despite their short length, microexons (here defined as ≤27 nucleotides (nt)) are conserved, highly included in neurons and harbor biological functions^35^. Using alternative exons (Methods) whose genes are expressed in the four major cell types, we calculated their *ψ* (percent spliced-in) values and considered the maximal ΔΨ (Methods) between these cell types for Cortex1 (Fig. 3a). Building on previous observations^35–37^, the most variable exons were enriched in microexons (<27 nt). However, highly variable exons with high *Ψ*s in neuronal or non-neuronal cell types were also enriched for exons ≤54 nt— that is, twice the maximal length for microexons and, albeit less pronounced, for ≤75 nt (Fig. 3b). Thus, cell-type-specific exon inclusion separates shorter exons from longer ones although far beyond the strict microexon definition. Cell-type-specific inclusion of disease-associated exons can pinpoint disease-implicated cell types. We, therefore, investigated published exons associated with schizophrenia^38^, ASD^35,39,40^ and ALS^41^ for inclusion variability across cell types. Separating our 5,855 alternative exons into schizophrenia-associated and non-schizophrenia-associated exons, we found no significance (two-sided Wilcoxon rank-sum test, *P* = 0.13) and only a 1.2-fold ratio between the two medians. Likewise, considering ALS, we found a fold change of ratio close to 1, albeit with a significant *P* value in one replicate. Thus, the schizophrenia-associated exons used here behave largely like random alternative exons in terms of cell-type-specific inclusion. ASD-associated exons, however, behaved differently. ASD-associated exons were considerably more variable across cell types (two-sided Wilcoxon rank-sum test, *P* < 2.22 × 10^−16^), with a 2.2-fold-higher median than non-ASD-associated alternative exons. The genes from which these disease-associated exons are derived were largely distinct for each disease considered and had no significant gene expression variability between the cell types (Supplementary Fig. 7a,b). Additionally, to control for previous observations regarding microexons in ASD, we excluded microexons from our comparative analysis, and the observation remained true (Fig. 3c). This variability of ASD-associated exons does not stem from inclusion in one specific cell type. Indeed, apart from many exons highly included in all four cell types, we observed two other groups: one exhibited high neuronal inclusion but low glial inclusion, and, conversely, a second showed high glial but low neuronal inclusion. More complicated cell-type-specific arrangements were observed less often, and these results can be extended to other broad cell types (Fig. 3d and Supplementary Fig. 7c).

Of the above 5,855 alternative exons, 586 were novel with respect to the GENCODE annotation (version 34) and had ≥10 overlapping reads in ≥1 cell type. The question of which exons should be included in state-of-the-art annotations is relevant^42^. To prioritize novel exons, we analyzed each exon’s variability (maxΔΨ; Methods) against its overall *Ψ* from all nuclei combined (termed ‘pseudo-bulk’) (Fig. 3e). We found four novel exon subsets: high variability and high inclusion (*n* = 6, top right); high variability but low inclusion (*n* = 20, top left); high inclusion but low variability (*n* = 30, bottom right); and low inclusion and low variability (*n* = 206, bottom left). Although all novel exons could be impactful, and the 0.5 cutoff is arbitrary, the first three categories suggest very high importance in at least one cell type (Fig. 3e). The above observations were broadly replicable in Cortex2 (Supplementary Fig. 7d–g). *CADM1* illustrates multiple highly cell-type-specific alternative exons in one gene (Fig. 3f). Three alternative exons are included more in astrocytes, oligodendrocyte precursor cells (OPCs) and oligodendrocytes than in both neuron types. Two alternative exons (Alt. exon 2 and Alt. exon 3; Fig. 3f) are very highly included in astrocytes. The inclusion in glia and ASD association of Alt. exon 3 motivates the exploration of its possible glial mis-regulation in ASD. In the event that new disease-associated exons are published, these can be explored on our interactive web portal (https://isoformatlas.com/).

### Combinations of transcript elements show distinct pairing rules

**Fig. 4:**
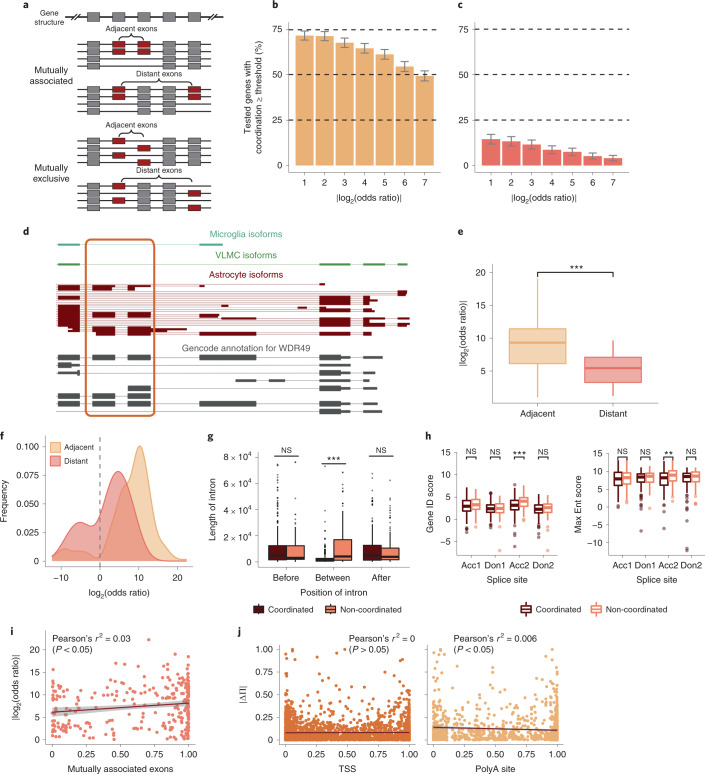
Coordination of adjacent and distant exon pairs. **a**, Schematic showing types of exon coordination patterns when considering two alternative exons (red). Mutual inclusion (top) and mutual exclusion (bottom) of distant and adjacent alternative exons. **b**, **c**, Bar plots showing percent of tested genes in pseudo-bulk with significant exon coordination for adjacent (**b**; *n* = 329) and distant (**c**; *n* = 173) exon pairs at various log-odds ratio cutoffs on the *x* axis. Error bars indicate s.e. of the point estimate. **d**, Region of adjacently coordinated exons for the *WDR49* gene. Each horizontal line indicates one transcript, colored by cell type; clustered blocks denote exons. Gray denotes annotated GENCODE transcripts. Orange box highlights the coordinated exons. **e**, Box plots of the |log-odds ratio| for significant genes on the *y* axis plotted against adjacent (*n* = 236) and distant (*n* = 25) exon pairs seen in **b** and **c** on the *x* axis. **f**, Density plot for the log-odds ratio for adjacent and distant exon pairs. **g**, Box plots of the length of introns flanking (before and after) and between pairs of adjacent exons. **h**, Splice site scores (left: GeneID; right: MaxEnt) for donor and acceptor splice sites for each exon in an adjacent pair. Color (**g**, **h**) indicates coordination status. **i**, Scatter plot of the |log-odds ratio| of coordination for exon pairs tested for association versus the minimum primate PhastCons score from the exon pair. **j**, Scatter plot of the ΔΠ versus the minimum PhastCons score among the TSSs (left) and polyA sites (right) associated with an exon. Regression lines (**i**, **j**) with 95% confidence interval obtained using the loess fit. *P* values (**e**, **g**, **h**) obtained from two-sided Wilcoxon rank-sum test. *P* values (**i**, **j**) from two-sided Pearson’s product moment correlation statistic. Significance: *P < 0.05; **P < 0.005; ***P < 0.001; NS, not significant. For box plots: center line, median; box limits, upper and lower quartiles; and whiskers, 1.5× interquartile range. VLMC, vascular lepotomeningeal cell.

We and others have investigated patterns of exon combinations; however, the frequency of different combination patterns remains unclear. Two exons may be paired non-randomly (that is, in a coordinated fashion) or randomly. The former can represent a tendency for mutual association or exclusion (Fig. 4a). When two exons within a transcript are coordinated (mutually associated/exclusive) in pseudo-bulk, we investigate if this is also true in ≥1 cell type (Supplementary Fig. 8a,b). Our testing strategy is similar to previous approaches and performs similarly^7,21,43^ (Supplementary Fig. 8c). For our analysis of exon coordination, we first considered alternative exon pairs. After false discovery rate (FDR) calculation, only one exon pair per gene was retained to avoid patterns representing few genes with many exon pairs (Methods). Among neighboring exon pairs, 71.4% of tested pairs, each represented by a 2 × 2 table, showed a significant association at FDR = 0.05 and |log-odds ratio | ≥ 1. By definition, this fraction decreases for higher log-odds ratios. However, even for a |log-odds ratio| ≥ 7, that is, a 128-fold enrichment of two of the exon combinations over the other two, ≥50% of exon pairs showed non-random pairing (Fig. 4b). For distant alternative exon pairs—that is, those with intervening exons, which we investigated previously^7,12,20^—this fraction was substantiaslly lower (Fig. 4c). An example of neighboring coordinated exons is the *WDR49* gene. Two neighboring coding exons are positively and perfectly coordinated—that is, all molecules include either both exons or none, whereas molecules with only one exon are not observed. In this case, coordination of both exons originates from an individual cell type, namely astrocytes (Fig. 4d). Adjacent coordinated alternative exons showed stronger coordination than distant coordinated exon pairs (Fig. 4e). Furthermore, distant exon pairs frequently show mutual exclusion coordination—that is, a negative log-odds ratio, whereas this is considerably less likely for adjacent exon pairs (Fig. 4f), which dominate our dataset. Compared to non-coordinated exon pairs, coordinated exon pairs were separated by smaller introns and had weaker acceptor strength for the second exon according to two splice site models^44,45^ (Fig. 4g,h). Similar observations arise for Cortex2 (Supplementary Fig. 9a–d). Consistent with adjacent mutually exclusive exons often exhibiting sequence homology^46^, and given that our adjacent coordinated exons are mostly mutually inclusive, we found almost no sequence similarity between these exon pairs. Given their tight coordination, we hypothesized that coordinated adjacent exon pairs would be highly evolutionarily conserved. We observed low significant correlation (Pearson’s *r*^2^ = 0.03, *P* = 0.004) between PhastCons scores^47^ of the less conserved mutually associated exon and coordination strength (Methods and Fig. 4i). Mutually exclusive adjacent exon pairs were too rare to investigate separately. Thus, evolutionarily recent exons have almost as tightly coordinated pairs as ancient exons. Similarly, we found little correlation between TSS/polyA site PhastCons scores and their coordination to internal exons (Fig. 4j).

### Coordination of exon pairs observed in bulk mostly stems from coordination in specific cell types

**Fig. 5:**
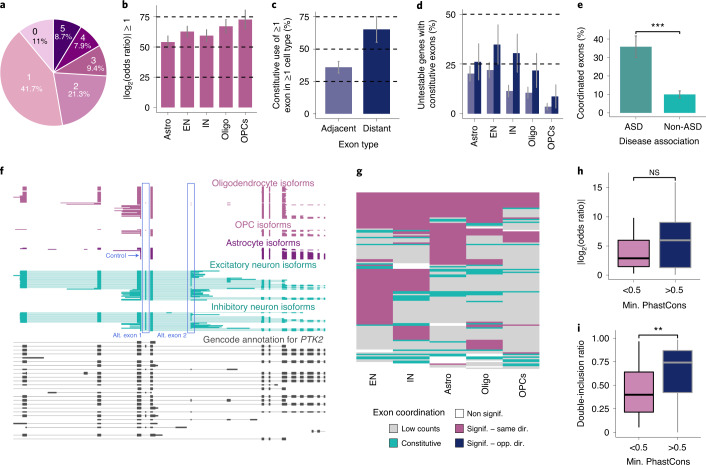
Exon coordination patterns are observable across multiple cell types. **a**, Pie chart of number of cell types where an exon pair is significant given significance in pseudo-bulk. **b**, Bar plot of percentage of tested exon pairs (one per gene) that were significantly coordinated. Cell type on the *x* axis (*n* = 98, 121, 101, 70 and 33). **c**, Bar plots of percentage of genes that are not testable in any cell type because at least one exon became constitutive. *x* axis values indicate adjacent (*n* = 114) or distant (*n* = 23). **d**, Bar plots of percentage of genes that are not testable in specific cell types because at least one exon became constitutive, colored by adjacent (*n* = 31, 31, 26, 26 and 22) or distant (*n* = 15, 15, 15, 15 and 14) exon pairs. **e**, Bar plot showing percent of distant coordinated exon pairs split by ASD association (*n* = 67 and 241). *P* value obtained from two-sided Fisher’s exact test. **b**–**e**, Error bars indicate s.e. of the point estimate. **f**, Distantly coordinated exons for the *PTK2* gene. Each horizontal line indicates one transcript, colored by cell type; clustered blocks indicate exons. Gray denotes annotated GENCODE transcripts. Blue boxes highlight coordinated exons, labeled Alt. exon 1 and Alt. exon 2. Control exon for qRT–PCR highlighted in blue. **g**, Heat map showing exon pairs that were testable in at least one cell type (*n* = 127). Exon pairs colored by significant coordination in same (pink) or opposite (blue) direction as pseudo-bulk, not significant (white), not testable in a particular cell type because of low counts (gray) or exons becoming constitutive (teal). **h**, Box plots of the |log-odds ratio| of excitatory neuron reads for tested exon pairs versus the minimum PhastCons score being <0.5 (*n* = 26) or >0.5 (*n* = 93). **i**, Box plots of the ratio of double inclusion of an exon pair to coordination (for excitatory neurons). *x* axis same as in **h**. For box plots: center line, median; box limits, upper and lower quartiles; and whiskers, 1.5× interquartile range. *P* values (**h**, **i**) calculated using the two-sided Wilcoxon rank-sum test. Significance: *P < 0.05; **P < 0.005; ***P < 0.001; NS, not significant. Astro, astrocyte; EN, excitatory neuron; IN, inhibitory neuron; Oligo, oligodendrocyte.

We then examined whether the coordination patterns at pseudo-bulk level were detected in at least one high-level cell type or whether they represent a heterogeneous mixture of homogeneous cell-type-specific patterns. Here, we considered excitatory neurons, inhibitory neurons, astrocytes, oligodendrocytes and OPCs as high-level cell types. Among the mostly adjacent coordinated pseudo-bulk exon pairs testable in ≥1 cell type, 89% were significantly coordinated in ≥1 cell type, meaning that the same patterns of coordination were observed in one or more cell types. More precisely, 41.7% were coordinated in one cell type, 21.3% in two cell types and 24% in three, four or five cell types (Fig. 5a). These observations were broadly conserved in Cortex2 (Supplementary Fig. 10a). In all five cell types investigated, ≥50% of testable (mostly adjacent) exon pairs showed significant coordination, but percentages varied among cell types. Indeed, for astrocytes, only 54.08% showed coordination, whereas, for oligodendrocytes and OPCs, 67.14% and 72.72% showed coordination, respectively (Fig. 5b and Supplementary Fig. 10b). Two distinct models can explain why an exon pair that is testable in pseudo-bulk is not testable in a cell type. First, read counts in a cell type, which are, by definition, lower than or equal to those in the pseudo-bulk, might simply be too low to allow for χ^2^ testing—a model purely technical in nature. Second, one or both of the exons might become constitutively included or skipped in the cell type (Methods and Supplementary Fig. 8b); this implies that the χ^2^ criterion for testability is violated—a model biological in nature. Distant alternative exon pairs are ~2-fold more likely to have ≥1 exon constitutively included/skipped in ≥1 cell type than adjacent alternative exons (Fig. 5c). This finding was replicated in each cell type separately, although non-overlapping 95% confidence intervals were observed only in excitatory neurons, inhibitory neurons and oligodendrocytes (Fig. 5d).

In addition to, and partially based on, our previous observation of ASD-associated exons being more variably spliced than others, we also found that pairs of ASD-related exons are highly coordinated. Indeed, ASD-associated exons are part of a distant coordinated exon pair more frequently than exons not associated with ASD (two-sided Fisher’s exact test, *P* = 1.82 × 10^−6^; Fig. 5e and Supplementary Fig. 10c). An example of distant coordinated ASD-associated exons with a strong cell-type-specific component is the *PTK2* gene, which encodes for FAK and influences axonal growth regulation and neuronal cell migration^48^. Two alternative microexons of 18 bp and 21 bp are highly included in excitatory and inhibitory neurons (co-inclusion score = 0.8 and 0.7; Methods) but are almost completely skipped in glial types (co-inclusion score = 0, 0.02 and 0, respectively, for astrocytes, oligodendrocytes and OPCs). We validated this highly cell-type-specific inclusion of these two exons using qRT–PCR (Methods and Supplementary Fig. 10d,e). Additionally, six of ten tryptic peptides obtained from ASD-associated exons that were detectable in mouse cell-type-specific proteome data^49^ showed the same cell-type-specific tendencies as the human exons (Methods and Supplementary Fig. 10f). This further motivates single-cell long-read investigations of ASD.

All significant cell-type-specific exon coordination values pointed in the same direction as in the pseudo-bulk. That is, coordination values for adjacent exon pairs observed in bulk reflect coordination in ≥1 cell type. Neurons and astrocytes clearly recapitulated more coordination events from the pseudo-bulk than oligodendrocytes and OPCs, likely owing to their higher nuclei numbers (Fig. 5g). Additionally, because of the strong tendency for mutual inclusion for adjacent exons, most molecules represent the mutually associated exons. In Cortex2, excitatory neurons dominated the genes that were significantly coordinated in bulk due to high excitatory neuron number in Cortex2 (Supplementary Fig. 10g).

Consistent with our pseudo-bulk observations, we found no significant association between exon conservation and coordination at cell type level (excitatory neurons as a representative cell type; Fig. 5h). In contrast to this observation, conservation was significantly associated with the inclusion of both alternative exons, an observation replicable in Cortex2 (Fig. 5i and Supplementary Fig. 10h,i).

### TSS–exon and exon–polyA site coordination often stems from constitutive use of variable sites in distinct cell types

**Fig. 6:**
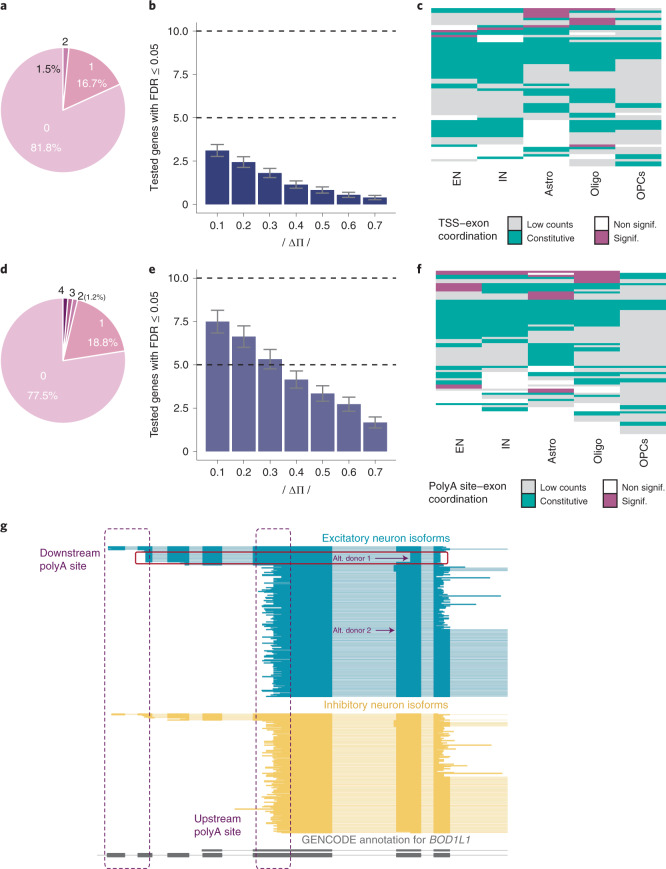
Exon–end site coordination is mediated by individual cell types. **a**, Pie chart of number of cell types where an exon–TSS pair is significant given significance in pseudo-bulk. **b**, Bar plot showing percent of tested genes in pseudo-bulk with significant exon–TSS coordination (*n* = 2,540) on the *y* axis and various Δ∏ cutoffs on the *x* axis. **c**, Heat map showing cell types as columns and exon–TSS pairs as rows (*n* = 66). Each element of the heat map is colored by whether the exon–TSS pair showed significant coordination (pink), was not significant (white) or was not testable because of low counts (gray) or because an exon or TSS became constitutively included in a cell type (teal). **d**, Pie chart indicating the number of cell types where an exon–polyA site pair is significant given the same testing conditions as in **a**. **e**, Bar plot (*n* = 1,615) showing exon–polyA site coordination as in **b**. **f**, Heat map showing cell types as columns and testable exon–polyA sites as rows (*n* = 80). Each element of the heat map is colored as in **c**. Error bars (**b**, **e**) indicate s.e. of the point estimate. **g**, Full-length transcript expression broken down by cell type for the *BOD1L1* gene. Each horizontal line is one transcript; clustered blocks indicate exons. Gray denotes annotated GENCODE transcripts. Purple boxes highlight region of interest. Astro, astrocyte; EN, excitatory neuron; IN, inhibitory neuron; Oligo, oligodendrocyte.

When tracing coordinated exon–TSS events into five major cell types, we observed considerably different behavior than that of adjacent exon pairs: in 82% of cases, significant coordination was not observed in any cell type, whereas, in ~17% and 1%, coordination was found in one and two cell types, respectively. Significance in ≥3 cell types, however, was never observed, and the overall proportion of genes exhibiting TSS–exon coordination was less than 5% at all investigated *Δ∏* cutoffs (Fig. 6a,b and Methods). Contrarily to adjacent exon pairs, constitutive use of one alternative site (TSS or exon) in a cell type occurred frequently and broadly consistently in all five cell types (Fig. 6c, teal). Exon–polyA site pairs were overall more consistent with the exon–TSS pairs than with exon–exon pairs in terms of how many individual cell types a coordination event was observed in, and the results were consistent in Cortex2 (Fig. 6d,e and Supplementary Fig. 11a–d). Likewise, constitutive inclusion/skipping of either the exon or polyA site in a cell type was observed far more often than for exon–exon pairs and slightly less than for exon–TSS pairs (Fig. 6f; compare with Figs. 6c and 5g). Coordination of polyA sites with exons is exemplified by *BOD1L1*. Two main polyA sites are observed. When the downstream polyA site is used, an upstream donor results in a shorter exon. Use of the upstream polyA site, however, mostly results in a longer exon. These observations are apparent in pseudo-bulk and in excitatory neurons. In inhibitory neurons, however, the longer exon is constitutively used, and coordination testing using χ^2^ statistics is impossible. In summary, the exon–polyA site coordination observed in the pseudo-bulk exists in excitatory neurons but not in other cell types (Fig. 6g).

## Discussion

Elucidating combination patterns of transcript elements—TSSs, exons and polyA sites—is necessary for a comprehensive understanding of biology, because these patterns define full-length isoforms carrying protein-coding information. To identify affected cell-type-specific splicing patterns in disease, it is paramount to first understand the combinations in healthy tissue, particularly of disease-associated exons. Moreover, brain region and cell-type-specific isoform expression might be critical to understanding the clinical relevance of deleterious variants of uncertain importance observed in patient genomes. To investigate these questions, we developed SnISOr-Seq (Fig. 1), an approach applicable to any single-nuclei RNA sequencing library. Although single-nuclei RNA sequencing is employed for many tissues, it is especially relevant for frozen samples, for which whole-cell isolation is difficult, a prominent example being the human brain.

SnISOr-Seq reveals single and combinatorial usage patterns of transcript elements. Consistent with previous reports^35–37^, we found that microexons (that is, exons ≤27 bp) show more variable inclusion across cell types than longer exons. However, exons considerably longer than 27 bp (up to ~75 bp depending on variability cutoff) also show high variability. ASD-associated exons, even when excluding microexons, show higher inclusion variability across the four major cell types than random alternative exons. In contrast, the trend for schizophrenia-associated or ALS-associated exons is substantially weaker or non-existent. In other words, although a fraction of ASD-associated exons exhibit similar inclusion in the four major cell types, a greater proportion show cell type specificity than for other diseases. The contrast between ASD and ALS/schizophrenia has the caveat that experiments to define disease-associated exons differ. However, should new exon–disease associations be identified, such exons can be queried against our data on our online interface (https://isoformatlas.com/). ASD-associated exons can have high glial, neuronal or uniform inclusion. The presence of both cell-type-biased and unbiased patterns implies that these exons are not well investigated by fluorescence-activated cell sorting (FACS) a single cell type. Single-cell investigations of exon pairing might be even more relevant for schizophrenia-associated and ALS-associated exons, which are no more variably included across cell types than background exons. These observations raise the fundamental question of whether, in disease, the inclusion of these disease-associated exons are altered in all cell types equally or whether their *Ψ* values change in particular cell types.

Ample research has investigated exon pairs, TSS–exon pairs and exon–polyA site pairs^7,21,26,28^. However, until now, a comparative analysis of these had been lacking in human brain. We found that adjacent exon pairs are combined more often and less randomly than distant pairs. In fact, most genes tested showed coordination of ≥1 adjacent exon pair. The gene fraction with coordinated exons could increase even further with deeper sequencing. Moreover, adjacent coordinated alternative exons are almost always mutually inclusive, whereas distant alternative exons exhibit more mutual exclusivity. TSS–exon pairing and exon–polyA site pairing show similar coordination to distant alternative exons but significantly less than adjacent exon pairs.

**Extended Data Fig. 1:**
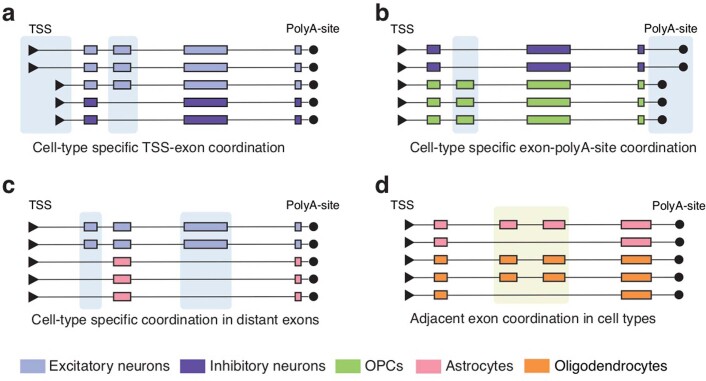
Models for cell-type specific coordination. **a**. Model for TSS-exon combinations. **b**. Model for exon-polyA-site combinations. **c**. Model for distant exon combinations. **d**. Model for adjacent exon combinations. Colors indicate different cell types. TSS: transcription start site; polyA-site: polyadenylation site; OPCs: oligodendrocyte precursor cells.

Considering cell-type-specific RNA expression, we found that three types of coordination, namely TSS–exon, exon–polyA site and distant exon–exon coordination, follow the same rule: these types of coordination are often observed at the pseudo-bulk level but are rarely traced into distinct human brain cell types. Often, they arise by one combination being expressed in one cell type and a different combination occurring in another. Thus, these types of coordination most often reflect the diversity of isoform expression distinguishing cell types (Extended Data Fig. 1a–c). Adjacent alternative exons, however, usually follow another pattern: whenever read counts suffice to trace coordination into specific cell types, we usually find one cell type, and often multiple cell types, in which this coordination occurs (Extended Data Fig. 1d).

Thus, when mutual association versus exclusivity and cell type specificity of coordination are considered, TSS–exon, polyA–site exon and distant exon pairs follow one model, whereas adjacent exon pairs follow a markedly different one. ASD-associated exons in distant exon pairs are more likely to be cell type specific and coordinated than non-ASD exons. Thus, splicing investigations of the brain in general and a deeper understanding of the role of these exons in neurological disease can benefit from further investigations enabled by SnISOr-Seq. The technologies developed here will also facilitate cross-species comparisons of cell-type-specific splicing.

## Methods

### Experimental model and subject details

#### Cortex samples for SnISOr-Seq

Two healthy human mid-frontal cortices used here were obtained from tissue banks maintained by the Center for Neurodegenerative Disease Research and the University of Pennsylvania Alzheimer’s Disease Core Center, according to institutional review board-approved protocols. Neither subject had pre-existing neurodegenerative or neurological disease. Postmortem intervals were 14 h for Cortex1 (age 68, male) and 6 h for Cortex2 (age 61, male). Tissues were flash-frozen and kept at −80 °C until processing.

#### Pre-frontal cortex samples for Illumina sequencing of bulk nuclei

Pre-frontal cortex (PFC) samples from two patients with Alzheimer’s disease used for Illumina sequencing were obtained from the Human Brain Tissue Bank (HBTB; Semmelweis University), which is a member of the BrainNet Europe II. HBTB’s activity is authorized by the Committee of Science and Research Ethic of the Ministry of Health Hungary (ETT TUKEB: 189/KO/02.6008/2002/ETT) and the Semmelweis University Regional Committee of Science and Research Ethic (32/1992/TUKEB), including human brain tissue sample removal, collecting and storing and applying for research. Human brain microdissection procedures were approved by the Regional and Institutional Committee of Science and Research Ethics of Scientific Council of Health (ethical license: 34/2002/TUKEB-13716/2013/EHR) and the Code of Ethics of the World Medical Association (Declaration of Helsinki). Genetic testing and international transportation samples were authorized by the Semmelweis University Regional Committee of Science and Research Ethics (34/2002/TUKEB). The postmortem interval was 6.5 h for PFC_S1 (age 93, female) and 5 h for PFC_S2 (age 81, male). In both cases, the tissue samples were microdissected from the dorsolateral PFC (middle frontal gyrus, Brodmann area 9). The micropunch procedure consisted of slicing the PFC into serial coronal sections, micropunching from both the surface and the deep (wall of the superior frontal sulcus) parts of the gyrus and collecting tissue pellets. Until processing, the brains were frozen and kept at −80 °C.

#### Fetal human samples for qRT–PCR validation

Neurons (Thy1^+^ cells) and astrocytes (HepaCAM^+^ cells) were isolated from fetal human brain tissue (*n* = 3, gestational weeks 19–20) using the immunopanning method^50^. The fetal human brain tissue samples were obtained with informed consent under a Stanford University institutional review board-approved protocol.

### Single-nuclei isolation and 10x Genomics 3′ library construction

Single-nuclei suspension was isolated from fresh-frozen human brain samples with modifications from a previous protocol^51,52^.

Next, ~30 mg of frozen tissue per sample was dissected in a sterile dish on dry ice and transferred to a 2-ml glass tube containing 1.5 ml of nuclei pure lysis buffer (MilliporeSigma, L9286) on ice. Tissue was completely minced and homogenized to nuclei suspension by sample grinding with Dounce homogenizers (Sigma, D8938-1SET) with 20 strokes with pestle A and 18 strokes with pestle B. The nuclei suspension was filtered by loading through a 35-µm-diameter filter and followed by centrifuging for 5 min at 600*g* and 4 °C. The nuclei pellet was collected and washed with cold wash buffer, which consisted of the following reagents: 1× PBS (Corning, 46-013-CM), 20 mM DTT (Thermo Fisher Scientific, P2325), 1% BSA (NEB, B9000S) and 0.2 U µl^−1^ of RNase inhibitor (Ambion, AM2682) for three times. After removing the supernatant from the last wash, nuclei were resuspended in 1 ml of 0.5 µg ml^−1^ of DAPI (Sigma, D9542) containing wash buffer to stain for 15 min. The nuclei suspension was prepared for sorting by filtering cell aggregates and particles out with a diameter of 35 µm. Nuclei were sorted to remove cell debris and fractured nuclei using the Sony MA900 sorter with FlowJo version 10 software (Supplementary Fig. 12a–c). These were collected by centrifuging for 5 min at 600*g* and 4 °C and then resuspended in wash buffer to reach a final concentration of 1 × 10^−6^ nuclei per milliliter after counting in trypan blue (Thermo Fisher Scientific, T10282) using a Countess II cell counter (Thermo Fisher Scientific, A27977).

10x Genomics 3′ library construction was performed by following the manufacturer’s instructions with single-nuclei suspension obtained from the last step. 10x Genomics 3′ libraries of Cortex1 and Cortex2 were loaded on an Illumina NovaSeq 6000 with PE 2 × 50 paired-end kits by using the following read length: 28 cycles Read1, eight cycles i7 index and 91 cycles Read2.

### Linear/asymmetric PCR steps to remove non-barcoded cDNA

The first round PCR protocol (95 °C for 3 min, 12 cycles of 98 °C for 20 s, 64°C for 30 s and 72 °C for 60 s) was performed by applying 12 cycles of linear/asymmetric amplification to preferentially amplify one strand of the cDNA template (30 ng of cDNA generated by using 10x Genomics Chromium Single Cell 3ʹ GEM kit) with primer ‘Partial Read1’, and then the product was purified with 0.8× SPRIselect beads (Beckman Coulter, B23318) and washed twice with 80% ethanol. The second round PCR is performed by applying four cycles of exponential amplification under the same condition with forward primer ‘Partial Read1’ and reverse primer ‘Partial TSO’, and then the product was purified with 0.6× SPRIselect beads and washed twice with 80% ethanol and eluted in 30 µl of buffer EB (Qiagen, 19086). Sequences of primers: Partial Read1 (5′-CTACACGACGCTCTTCCGATCT-3′) and Partial TSO (5′-AAGCAGTGGTATCAACGCAGAGTACAT-3′). KAPA HiFi HotStart PCR Ready Mix (2×) (Roche, KK2601) was used as polymerase for all the PCR amplification steps in this paper, except for the 10x Genomics 3′ library construction part.

### Exome capture to enrich for spliced cDNA

Exome enrichment was applied to the cDNA purified from the previous step by using probe kit SSELXT Human All Exon V8 (Agilent, 5191-6879) and the reagent kit SureSelectXT HSQ (Agilent, G9611A), according to the manufacturer’s manual. First, the block oligo mix was made by mixing an equal amount (1 µl of each per reaction) of primers Partial Read1 (5′-CTACACGACGCTCTTCCGATCT-3′) and Partial TSO (5′-AAGCAGTGGTATCAACGCAGAGTACAT-3′) with the concentration of 200 ng µl^−1^ (IDT), resulting in 100 ng µl^−1^. Next, 5 µl of 100 ng µl^−1^ cDNA diluted from the previous step was combined with 2 µl of block mix and 2 µl of nuclease free water (NEB, AM9937), and then the cDNA block oligo mix was incubated on a thermocycler under the following conditions to allow block oligo mix to bind to the 5′ end and the 3′ end of the cDNA molecule: 95 °C for 5 min, 65 °C for 5 min and 65 °C on hold. For the next step, the hybridization mix was prepared by combining 20 ml of SureSelect Hyb1, 0.8 ml of SureSelect Hyb2, 8.0 ml of SureSelect Hyb3 and 10.4 ml of SureSelect Hyb4 and kept at room temperature. Once the reaction reached to 65 °C on hold, 5 µl of probe, 1.5 µl of nuclease-free water, 0.5 µl of 1:4 diluted RNase Block and 13 µl of the hybridization mix were added to the cDNA block oligo mix and incubated for 24 h at 65 °C. When the incubation reached the end, the hybridization reaction was transferred to room temperature. Simultaneously, an aliquot of 75 µl of M-270 Streptavidin Dynabeads (Thermo Fisher Scientific, 65305) were prepared by washing three times and resuspended with 200 µl of binding buffer. Next, the hybridization reaction was mixed with all the M-270 Dynabeads and placed on a Hula mixer for 30 min at room temperature. During the incubation, 600 µl of wash buffer 2 (WB2) was transferred to three wells of a 0.2-ml PCR tube and incubated in a thermocycler on hold at 65 °C. After the 30-min incubation, the buffer was replaced with 200 µl of wash buffer 1 (WB1). Then, the tube containing the hybridization product bound to M-270 Dynabeads was put back into the Hula mixer for another 15-min incubation with low speed. Next, the WB1 was replaced with WB2, and the tube was transferred to the thermocycler for the next round of incubation. Overall, the hybridization product bound to M-270 Dynabeads was incubated in WB2 for 30 min at 65 °C, and the buffer was replaced with fresh pre-heated WB2 every 10 min. When the incubation was over, WB2 was removed, and the beads were resuspended in 18 µl of nuclease-free water and stored at 4 °C. Next, the spliced cDNA, which bound with the M-270 Dynabeads, was amplified with primers Partial Read1 and Partial TSO by using the following PCR protocol: 95 °C for 3 min, 12 cycles of 98 °C for 20 s, 64 °C for 60 s and 72 °C for 3 min. The amplified spliced cDNA was isolated from M-270 beads as supernatant and followed by a purification with 0.6× SPRIselect beads.

### Library preparation for PacBio

HiFi SMRTbell libraries of Cortex1 and Cortex2 were constructed according to the manufacturer’s manual by using SMRTbell Express Template Prep Kit 2.0 (PacBio, 100-938-900). For both samples, ~500 ng of cDNA obtained by performing LAP-CAP from the previous step was used for library preparation. The library construction includes DNA damage repair (37 °C for 30 min), end-repair/A-tailing (20 °C for 30 min and 65 °C for 30 min), adaptor ligation (20 °C for 60 min) and purification with 0.6× SPRIselect beads.

### Library preparation for ONT

For both samples, ~75 fmol cDNA processed through LAP-CAP underwent ONT library construction by using the Ligation Sequencing Kit (ONT, SQK-LSK110), according to the manufacturer’s protocol (Nanopore Protocol, Amplicons by Ligation, version ACDE_9110_v110_revC_10Nov2020). The ONT library was loaded onto a PromethION sequencer by using PromethION Flow Cell (ONT, FLO-PRO002) and sequenced for 72 h. Base-calling was performed with Guppy by setting the base quality score >7.

### RNA extraction and cDNA synthesis for Illumina short-read sequencing

RNA was extracted from the single-nuclei suspension containing 300,000 nuclei by using the RNeasy Mini Kit (74104), which involved on-column gDNA digestion before RNA elution. cDNA was synthesized and amplified with NEBNext Single Cell/Low Input cDNA Synthesis & Amplification Module (E6421S) by following the manufacturer’s protocol.

### Short-read library preparation and sequencing with Illumina

With 100 ng of cDNA input per sample, Illumina library prep was conducted with the Illumina DNA Prep (M) Tagmentation Kit (20018704) and IDT for Illumina Nextera DNA Unique Dual Indexes Set D (20027216), according to the manufacturer’s manual. Libraries were loaded on an Illumina NextSeq 500 with 2 × 150 bp Mid Output Kit by using the following read length: ten cycles Read1, ten cycles i7 index and 76 cycles Read2.

### Validation of exon coordination in PTK2 using qRT–PCR

Neurons (Thy1^+^) and astrocytes (HepaCAM^+^) were isolated from fetal human brain tissue (*n* = 3, gestational weeks 19–20) using immunopanning^50^. All fetal human brain tissue samples were obtained with informed consent under a Stanford University institutional review board-approved protocol. RNA was extracted from about 5 million purified neurons or astrocytes with QIAzol Lysis Reagent (Qiagen, 79306). First-strand cDNA was reverse transcribed using SuperScript IV Reverse Transcriptase (Invitrogen, 18090050). RT–qPCR was performed using 15 ng of cDNA as template per sample, validated primers (see below) and PowerUp SYBR Green Master Mix (Applied Biosystems, A25742) on a QuantStudio 3 Real-Time PCR System (Thermo Fisher Scientific). Primers for RT–qPCR were designed by using Primer-BLAST (https://www.ncbi.nlm.nih.gov/tools/primer-blast/) and synthesized by Thermo Fisher Scientific. The primers either targeting the control exons or spanning alternatively spliced PTK2 exon 1 or exon 2 are listed below. The specificity of each primer pair was validated through the observation of a single band on an electrophoresis gel under a fixed melting temperature of PCR condition. The efficiency of each primer pair was evaluated as 85–115%. Comparisons were made using the comparative *C*_T_ method^53^ and normalized to neurons, shown as fold change in Supplementary Fig. 10c.

### RT–qPCR primers

PTK2_Alternative_exon1

5′-CACGCTGTCCGAAGTACAGT-3′ and 5′-ATGGAATAGATGAAGCCAGGG-3′

PTK2_Alternative_exon2

5′-AACCGCCAAAGCTGGATTCT-3′ and 5′-TGAAATTAGTGGGGACGAAACA-3′

PTK2_Mutual_exons (control)

5′-GCCTTCTCCAATACATCGTCCA-3′ and 5′-GATACTTACACCATGCCCTCA-3′

### Proteomic validation of cell-type-specific coordination of ASD-associated exons

Mass spectrometry raw data from the ProteomeXchange dataset PXD001250 were searched against the UniProt mouse proteome (7 March 2021; 63,682 entries) with MaxQuant^49^ 2.0.3.0 using default settings. We normalized the peptide abundance matrix (label-free quantification) by median sample abundance. Relative abundances in neurons, astrocytes and oligodendrocytes were compared between exon Ψ (PSI) and corresponding peptide abundances (proteomics) using the subset of tryptic peptides from an in silico digest of the exon sequences that were also identified and quantified in the proteomics dataset. Peptides that ambiguously map to multiple genes in an in silico digest of the UniProt mouse proteome were discarded. For both—the Ψ values and the proteomics peptide abundances (mean over replicates)—we set a relative abundance threshold at 95% (of maximum abundance over cell types) to define their respective enriched cell type(s) and subsequently tested for overlap between both data sources.

### Data processing and quality control for single-cell short-read analysis

The 10x Cell Ranger pipeline (version 3.1.0) was run on raw Illumina sequencing data to obtain single-cell expression matrices that were analyzed using Seurat version 3.1.1 (ref. ^31^). For both samples, nuclei that had unique gene counts of >7,500 or <200 or >4% mitochondrial gene expression were discarded. This yielded 7,314 nuclei for Cortex1 and 6,486 nuclei for Cortex2. UMI numbers and mitochondrial gene expression percentages were regressed from each nucleus, and the matrix was log-normalized and scaled to 10,000 reads per cell. Next, we clustered cells using the Louvain algorithm, setting the resolution parameter to 0.6. We performed both t-distributed stochastic neighbor embedding (tSNE) and uniform manifold approximation and projection (UMAP) non-linear reduction techniques. Cell types were assigned by identifying canonical marker genes for each cluster^13,54–56^. This cell type annotation was confirmed by aligning to the Allen Brain Atlas human cortical data^13^.

### Alignment of PacBio long-read data

Using default SMRT-Link parameters, we performed circular consensus sequencing (CCS) with IsoSeq3 with the following parameters: maximum subread length 14,000 bp, minimum subread length 10 bp and minimum number of passes 3.

Long-read CCS fastq sequences with PacBio were mapped and aligned to the reference genome (GRCh38) using STARlong and parameters described previously^33^

### In silico simulation of poly(dT) and random hexamer priming

Using GENCODE version 35 transcripts and ten copies per transcript, we simulated cDNA synthesis: introns were retained with *P* = 0.15, and 30 As were added to each transcript, which were cut into 2-kb fragments and shorter ends, with 1.9-kb mean resulting fragment size. Each fragment was then classified as (1) 3′-end fragment (with polyA tail) or (2) internal fragment (without polyA tail). For both types, random hexamer priming was simulated by choosing a random (uniform) position along the transcript. The sequence to the right of that position was kept as a sequenced molecule, and the remainder was discarded. For both types, poly(dT) priming was simulated by choosing the longest A-rich sequence with ≥8 As in a 10-bp window. The fragment to the right of the A-rich sequence was kept as the sequenced molecule, and the remainder was discarded. Note that more stringent criteria (≥9 As) would lead to more fragments being lost. These sequenced molecules were then mapped back to the annotation, and the fraction of covered transcript was reported.

### Alignment of ONT long-read data

Long reads sequenced on the ONT PromethION were mapped using minimap2 (version 2.17-r943-dirty) using the previously described parameters^33^.

### Calculation of on-target rate

For both long-read technologies, the on-target rate was calculated using the ‘intersect’ function from BEDTools (version 2.27.0) with this definition:

$$On-target\,rate = \frac{{No.\,of\,mapped\,reads\,that\,overlap\,annotated\,exons}}{{Total\,no.of\,mapped\,reads}}$$

### Calculation of normalized transcript coverage

Normalized transcript coverage was calculated using the ‘CollectRnaSeqMetrics’ function from Picard tools (version 2.25.7). A ‘refFlat’ gene annotation file was downloaded from http://hgdownload.cse.ucsc.edu/goldenPath/hg38/database/refFlat.txt.gz.

### Subsampling of sequencing libraries

Reads were subsampled using the ‘sample’ command from seqtk (version 1.3-r106).

### Calculation of a per-read exon ratio

The expected number of exons per GENCODE gene was obtained by counting exons of each transcript and averaging for all annotated transcripts. Subsequently, the observed exons per read were divided by the expected number, yielding a ratio for each read.

### In silico simulation of cDNA fragmentation

SnISOr-Seq long reads were truncated in silico so that a random number of 3′ nucleotides remained (normal distribution, mean = 250 and s.d. = 50) to simulate cDNA fragmentation. Then, 76 bp from the 5′ end of the remaining fragment were isolated to simulate the 76 bp R2 of the 10x Illumina library. Normalized sequencing coverage was then calculated.

### Barcode detection and identification of unique molecules from PacBio data

Cellular barcodes (16 nt) were detected using the ‘GetBarcodes’ function in scisorseqr^33^ (version 0.1.2). Given PCR duplication, one transcript per molecule—that is, barcode+UMI+gene—was chosen for analysis.

### Barcode detection for long-read transcripts obtained from ONT

Perfect matching barcodes were obtained similarly to the PacBio reads, however with some tolerance for sequencing errors, using the mapping information per read with white-listed UMIs as done previously^57^ with modifications:

- For each Illumina-sequenced UMI, all barcode–UMI 28mers were grouped by gene as a reference set.
- For every mapped ONT read, we compared only to the reference list for that gene.
- Sliding windows identified barcode–UMI candidates allowing ≤1 mismatch in the first 22 bp of each reference 28mer. We then allowed only ≤2 mismatches in the 28mer.

These steps were performed using a custom script.

### Identification of unique molecules from ONT data

Given the ONT error rate, reads were more likely to undergo ‘molecule inflation’—that is, errors could result in one UMI being perceived as two different ones. To combat this, we proceeded as follows:

- Reads were grouped by barcode–UMI–gene and ordered by frequency.
- The Levenshtein distance (LevD) to the nearest barcode–UMI pair from the same gene was obtained.
- If LevD = 0, it was retained as an Illumina-confirmed molecule.
- If LevD = 1 or 2, the 28-bp sequence was corrected to match the Illumina reference, and, if the barcode–UMI–gene triplet was novel with respect to ONT data, it was retained.
- If LevD > 3 and the edit distance to any other already accepted UMI was >5, the molecule was considered novel and retained.
- If LevD > 3 but the edit distance to any other already accepted UMI was 1 or 2, this UMI was corrected to the accepted UMI.

Following this sequencing error correction, only one read per barcode–UMI–gene triplet was retained. These steps were performed using a custom script.

### Assigning TSS and polyA site to reads

We assigned the closest published TSS within 50 bp of the 5′ end of the read mapping^58^ as previously done^33^. Likewise, we assigned the closest published polyA site within 50 bp of the 3′ end of the read mapping^59^.

### PhastCons scores for exons, TSSs and polyA sites from 17 primates

PhastCons scores for 16 primate genomes aligned to the human genome were obtained from the UCSC website^47,60^. The scores were averaged over internal exons, TSSs and polyA sites using the bigWigAverageOverBed script from the UCSC Utilities package.

### Disease-associated exons for ASD, schizophrenia and ALS

ASD-associated exons (*n* = 3,482) were summarized from two studies and one review: 1,776 skipped exons from a comparison of ASD cases with controls (*P* < 0.05)^39^, 1,723 neural regulated alternatively spliced exons from ASD brains^35^ and 33 microexons associated with ASD and characterized functionally^40^. Schizophrenia-associated exons that were classified as alternative exon skipping events covering 1,107 exons were collected^38^. The list of 506 ALS-associated cassette exons was identified by comparing *C9orf72* ALS brains with control brains^41^.

### Alternative exon counting and categorization

Using all exons appearing as internal exon in a read, we calculated:

1. The number of long-read UMIs containing this exon with identity of both splice sites: *X*_*in*_
2. The number of long-read UMIs assigned to the same gene as the exon, which skipped the exon and ≥50 bases on both sides: *X*_*out*_
3. The number of long-read UMIs supporting the acceptor of the exon and ending on the exon: *X*_*acc*_*In*_
4. The number of long-read UMIs supporting the donor of the exon and ending on the exon: *X*_*don*_*In*_
5. The number of long-read UMIs overlapping the exon: *X*_*tot*_

Non-annotated exons with one or two annotated splice sites, ≥70 bases of non-exonic (in the annotation) bases, were excluded as intron retention events or alternative acceptors/donors.

We then calculated

- $${\Psi}_{overall} = \frac{{X_{in} + X_{acc\_In} + X_{don\_In}}}{{X_{in} + X_{acc\_In} + X_{don\_In} + X_{out}}}$$
- $${\Psi}_{acceptor} = \frac{{X_{in} + X_{acc\_In}}}{{X_{in} + X_{acc\_In} + X_{out}}}$$
- $${\Psi}_{donor} = \frac{{X_{in} + X_{don\_In}}}{{X_{in} + X_{don\_In} + X_{out}}}$$

If

- $$0.05 \le {\Psi}_{condition} \le 0.95\,where\,condition \in \{ overall,acceptor,donor\}$$
- $$\frac{{X_{in} + X_{acc\_In} + X_{don\_In} + X_{out}}}{{X_{tot}}} \ge 0.8$$

the exon was kept.

We then calculated the Ψ_*overall*_ for each cell type from all long-read UMIs for that cell type if, and only if, *X*_*tot*_ ≥ 10 for the exon and cell type in question. Otherwise, Ψ_*overall*_ for the exon and cell type was set to ‘NA’.

### Exon variability analysis

For each replicate, we defined a set of alternative exons that met each of the following criteria: (1) ≥10 supporting reads (inclusion + exclusion) in the pseudo-bulk; (2) 0.05 < *Ψ* < 0.95 at the pseudo-bulk level; and (3) intron retention events were excluded. These steps yielded 5,855 (Cortex1) and 5,273 (Cortex2) alternative exons. We defined a subset of alternative exons with ≥10 supporting reads in each of four major cell types (excitatory neurons, inhibitory neurons, astrocytes and oligodendrocytes). We divided alternative exons into three variability categories: (1) (max*Ψ* − min*Ψ*) ≤ 0.25; (2) 0.25 < (max*Ψ* − min*Ψ*) ≤ 0.75; and (3) (max*Ψ* − min*Ψ*) > 0.75. For each category, we plotted the exon length density using ggplot2. Then, for each disease, we compared disease-associated exons with all other alternative exons for inclusion variability. Microexons were defined as exons with a length of ≤27 bp. Novel exons were defined as exons that are not described in the GENCODE version 34 annotation. To define a subset of novel exons that show high inclusion and/or high cell type variability, we plotted (max*Ψ* − min*Ψ*) against pseudo-bulk *Ψ* and fit a loess curve to the data.

### Gene variability analysis

Genes with disease-associated exons were isolated. log-normalized expression (transcripts per million (TPM)) values were obtained from the short-read 10x data. Variability per gene was defined as the minimum value across the broad cell types considered subtracted from the maximum value. *P* values were obtained by a two-sided Wilcoxon rank-sum test.

### Testing for exon coordination

Testing for exon coordination can be done at the pseudo-bulk level or at the cell type level. For every exon pair passing the criteria for sufficient depth, a 2 × 2 matrix of association for a given sample—that is, cell type or pseudo-bulk—was generated. This matrix contained counts for inclusion of both exons (in–in), inclusion of the first exon and exclusion of the second (in–out), exclusion of the first exon and inclusion of the second (out–in) and exclusion of both exons (out–out).

The co-inclusion score of an exon was defined as the double inclusion (in–in) divided by the total counts for that exon pair. An exon pair that was deemed ‘coordinated’ was assessed using the *χ*^2^ test of association. The effect size was calculated as the |log_10_(odds ratio)|. The odds ratio was calculated by setting 0 values to 0.5 and dividing the product of double inclusion and double exclusion by the product of single inclusion—that is, [(in–in) × (out–out)] / [(in–out) × (out–in)]. Finally, we used a Benjamini–Yekutieli correction for multiple testing and reported the FDR value.

### Conservation analysis for exon pairs

PhastCons scores from primates were obtained as described above. For every gene used in the pseudo-bulk analysis, the exon pairs with the smallest |log_10_(odds ratio)| were retained. The minimum PhastCons score for each pair was extracted and plotted against the absolute value of the log-odds ratio.

### Cell-type-specific conservation for exon pairs

Exon coordination count data were split by cell type, including astrocytes, excitatory neurons, inhibitory neurons, oligodendrocytes or OPCs. Together with the log-odds ratio, we calculated an exon inclusion ratio, which is defined as the number of times both exons pairs are included in the sequencing data (in–in) divided by the sum of the in–in and out–out counts per exon pair. The minimum PhastCons value for each exon pair was selected and placed into two groups ([0,0.5] and [0.5,1]). We then plotted these two groups against the |log_10_(odds ratio)| and the exon inclusion ratio as box plots.

### Obtaining counts for exon–end site combinations

We obtained counts for exon–TSS and exon–polyA site combinations using a custom script. Specifically, per sample we counted the number of reads assigned to a given TSS and divided them into reads including a particular exon or skipping the exon. We proceeded similarly for exon–polyA site pairs. Only genes with ≥25 reads were used for further analysis.

### Testing for exon–end site coordination

Testing for exon–end site coordination (here, with a *χ*^2^ test) can be done either in pseudo-bulk or in each cell type. For each test, a *n* × 2 matrix per internal exon was generated, with the *n* TSS forming rows and inclusion and exclusion counts forming columns. An exon–TSS pair was tested only if the TSS was upstream of the intron preceding the exon, and the read extended to beyond the end of the following intron. For effect size, we used *Δ∏* (the maximum change in exon inclusion between reads associated to distinct TSS). Finally, we used a Benjamini–Yekutieli correction for multiple testing and reported the FDR value. We proceeded similarly for exon–polyA sites.

### The χ^2^ criterion and testability

To categorize exon pairs or exon–end site pairs as testable, we employed the following metrics. For each matrix *M* with elements m_ij_,

- The expected value for each element m_ij_ was defined as $\frac{{\mathop {\sum }\nolimits_{k = 1}^i m_{kj} \cdot \mathop {\sum }\nolimits_{k = 1}^j m_{ik}}}{{{\sum} M }}$.
- If the expected value in 80% (rounded to nearest integer) of elements is ≥5, and the expected value of all elements is ≥1, the *χ*^2^ criterion is met, and the *P* value is calculated.
- If the median expected value is <5 in any row or any column, then the RNA variable (that is, TSS, exon or polyA site) in that row or column is said to be constitutive.
- If none is met, we classify them as ‘low counts’.

### Conservation analysis for exon–end site pairs

PhastCons scores for all TSSs were extracted as described above. For every gene in the pseudo-bulk analysis, the TSS–exon pair with the smallest Δ∏ was chosen. For such exons, PhastCons scores of the associated TSSs were sorted by value. The TSS with the minimum PhastCons score was reported for that exon, and the Pearson’s product–moment correlation between the PhastCons score and Δ∏ for that TSS–exon pair was calculated. Similar analysis was conducted for the exon–polyA site pairs.

### Reporting Summary

Further information on research design is available in the Nature Research Reporting Summary linked to this article.

## Online content

Any methods, additional references, Nature Research reporting summaries, source data, extended data, supplementary information, acknowledgements, peer review information; details of author contributions and competing interests; and statements of data and code availability are available at 10.1038/s41587-022-01231-3.

## Supplementary information

Supplementary Information Supplementary Figs. 1–12 and Supplementary Table 1

Reporting Summary

## Data Availability

All data used for this study are publicly available in the Gene Expression Omnibus under accession number GSE178175. All data supporting the findings of this study are provided within the paper and its Supplementary Information. Source data for the main figures can be found at https://github.com/noush-joglekar/sn-code.

## Code availability

The source code generated for this paper is publicly available at https://github.com/noush-joglekar/sn-code.
