# Supplementary material for: Single-nuclei isoform RNA sequencing unlocks barcoded exon connectivity in frozen brain tissue
Source: Nat Biotechnol. 2022 Mar 7;40(7):1082–92. doi: 10.1038/s41587-022-01231-3 (PMC9287170; doi:10.1038/s41587-022-01231-3)
Supplement: Supplementary file 1 — Supplementary Figs. 1–12 and Supplementary Table 1 [file 41587_2022_1231_MOESM1_ESM.pdf]

nature portfolio

https://doi.org/10.1038/s41587-022-01231-3

Supplementary information

Single-nuclei isoform RNA sequencing unlocks barcoded exon connectivity in frozen brain tissue

In the format provided by the authors and unedited

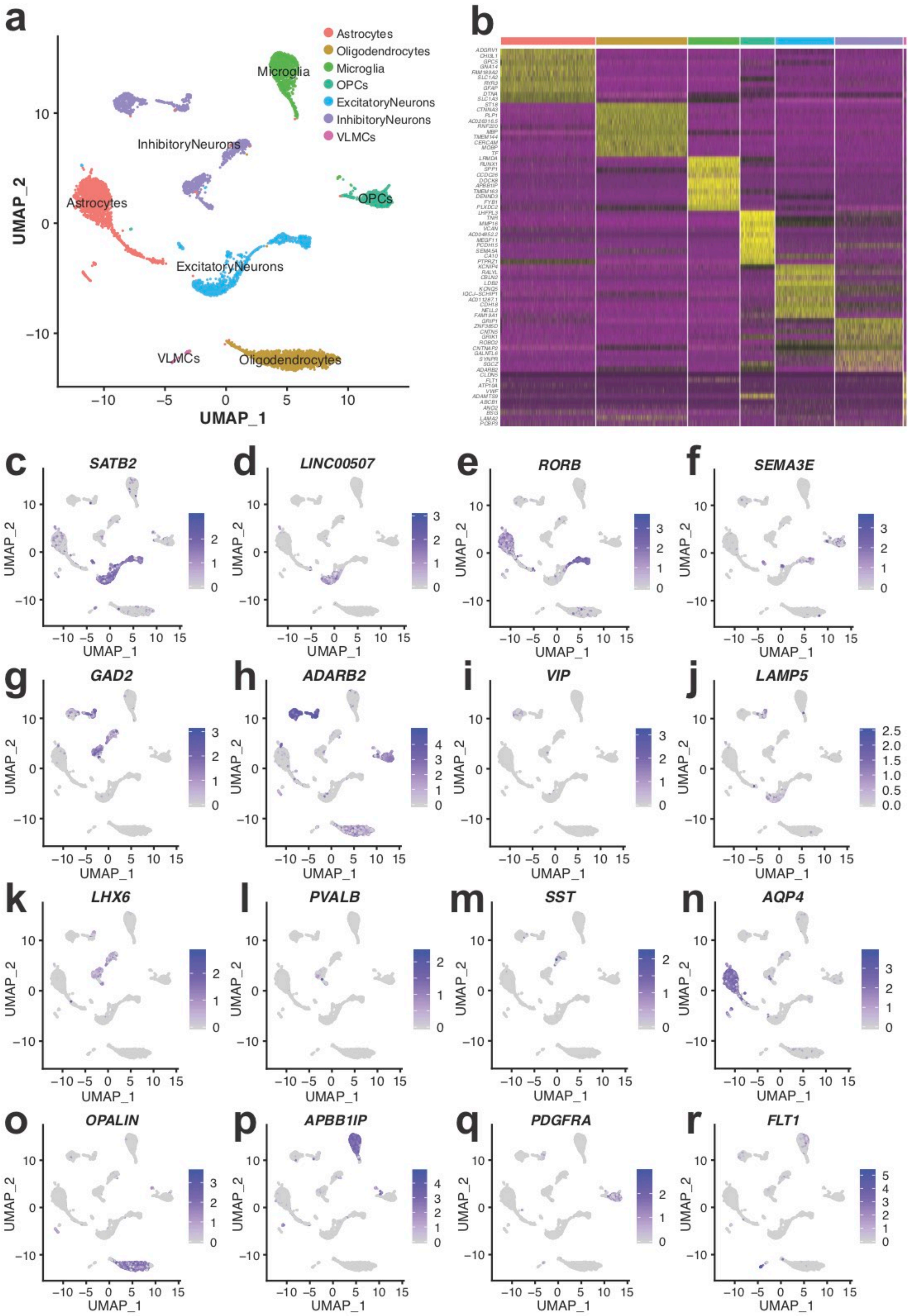
a
Astrocytes
Oligodendrocytes
Microglia
OPCs
ExcitatoryNeurons
InhibitoryNeurons
VLMCs
UMAP_1
UMAP_2
b
c SATB2
d LINC00507
e RORB
f SEMA3E
g GAD2
h ADARB2
i VIP
j LAMP5
k LHX6
l PVALB
m SST
n AQP4
o OPALIN
p APBB1IP
q PDGFRA
r FLT1

Supplementary Figure 1: Cell type assignment for frontal cortex single-nuclei sample. a. UMAP plot of Cortex1 sample with clusters labelled by cell type annotation. **b.** Heatmap of gene expression of marker genes that are enriched in each cluster depicted in A, with purple and yellow denoting low and high expression respectively. Each row corresponds to a gene while each column corresponds to a single nucleus. Nuclei are clustered by cell type, and annotation for each cell type is at the top. **c-r.** Normalized gene expression for the indicated marker genes projected onto the UMAP plot.

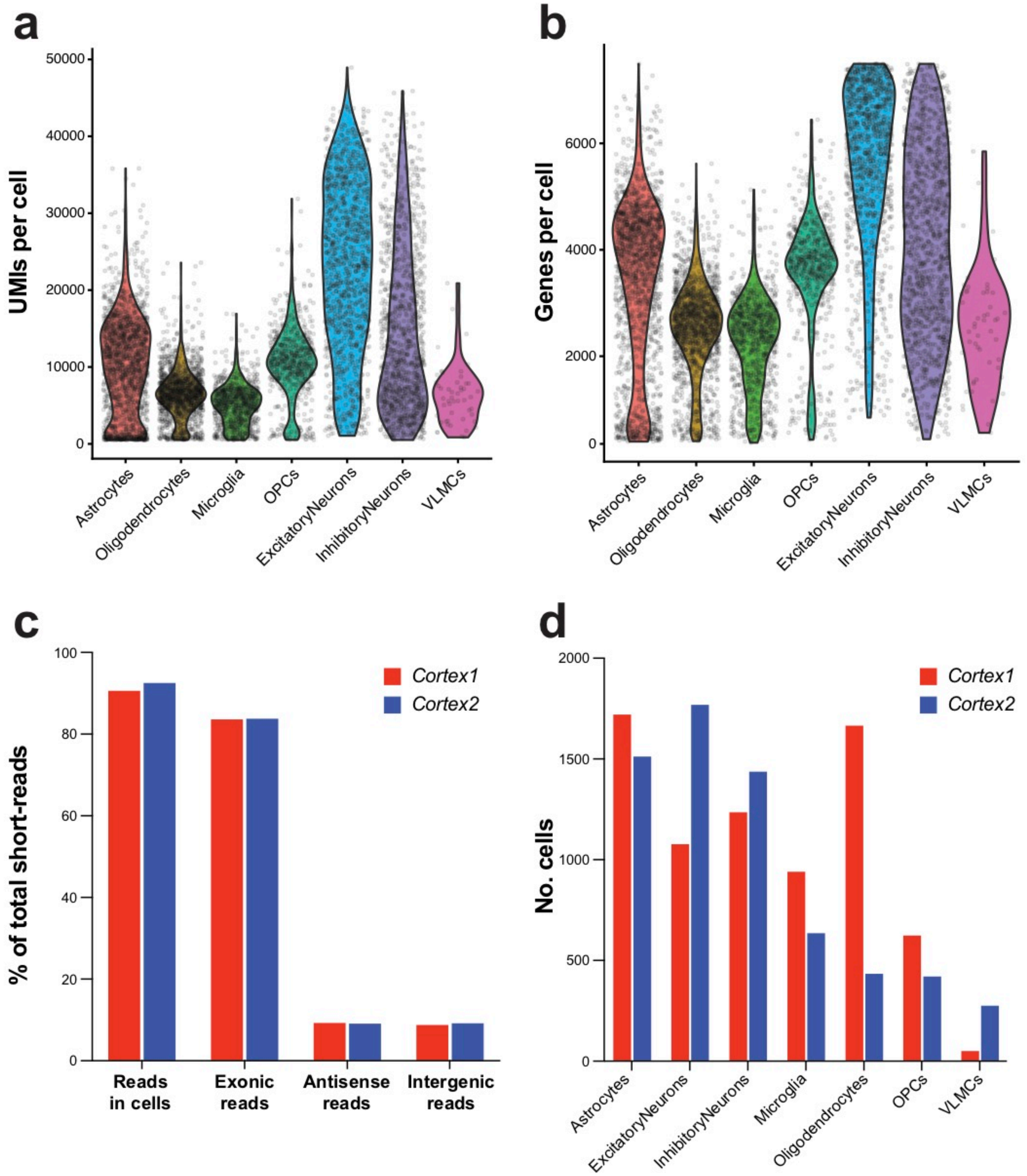

Supplementary Figure 2: Short-read sequencing statistics. a-b. Violin plots depicting UMIs (a) and genes (b) sequenced per single nucleus broken down by cell type which is indicated on the X-axis. **c.** Bar plot of the percentage of total sequenced reads assigned to each of the metrics defined on the X-axis. Color of bar indicates the sample. **d.** Bar plot of the number of single nuclei assigned to each cell type indicated on the X-axis. Color of bar indicates the sample.

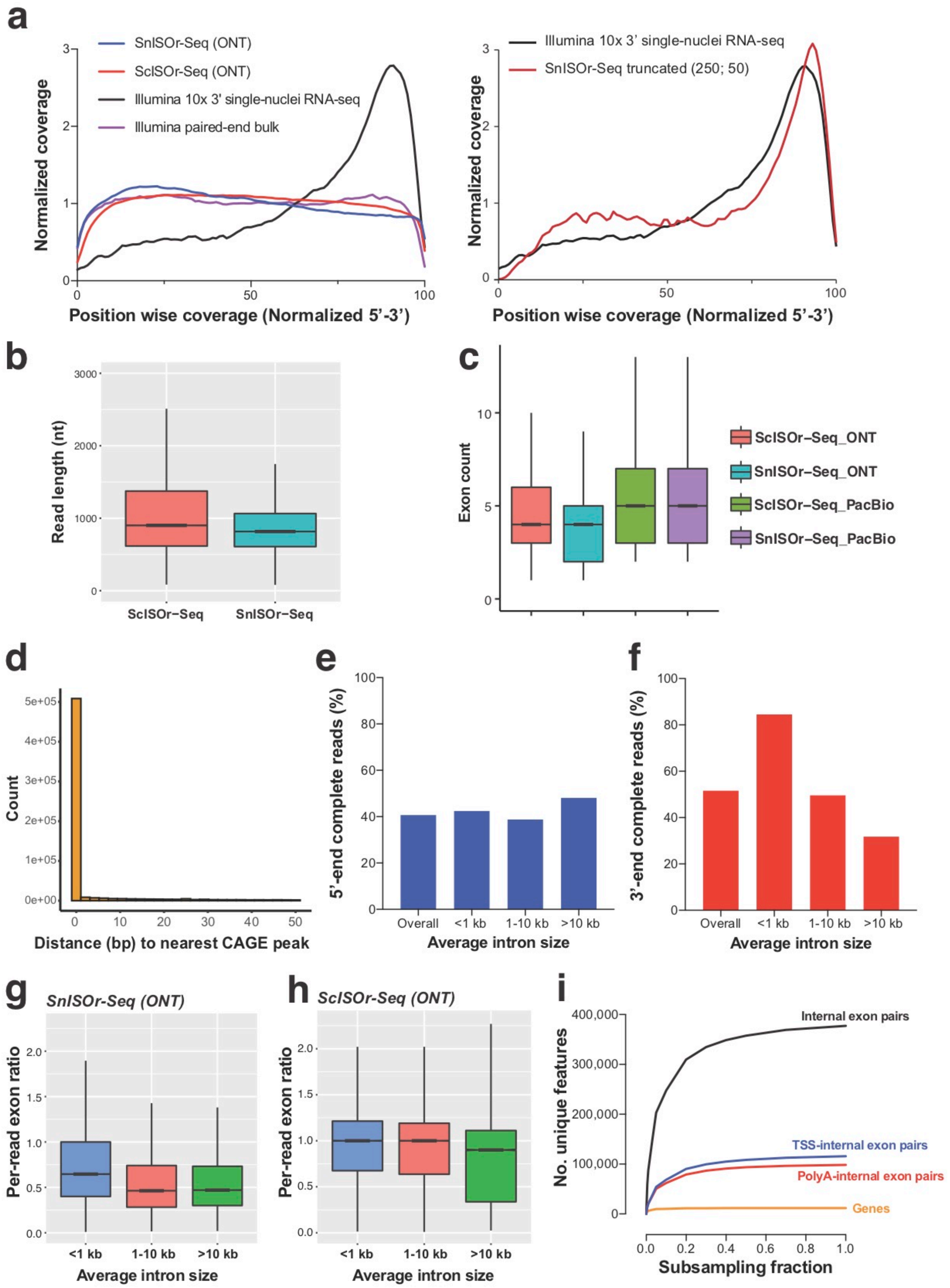

a
SnISOr-Seq (ONT)
ScISOr-Seq (ONT)
Illumina 10x 3' single-nuclei RNA-seq
Illumina paired-end bulk
Normalized coverage
Position wise coverage (Normalized 5'-3')
Illumina 10x 3' single-nuclei RNA-seq
SnISOr-Seq truncated (250; 50)
b
Read length (nt)
ScISOr−Seq
SnISOr−Seq
c
Exon count
ScISOr−Seq_ONT
SnISOr−Seq_ONT
ScISOr−Seq_PacBio
SnISOr−Seq_PacBio
d
Count
Distance (bp) to nearest CAGE peak
e
5'-end complete reads (%)
Overall
<1 kb
1-10 kb
>10 kb
Average intron size
f
3'-end complete reads (%)
Average intron size
g
SnISOr-Seq (ONT)
Per-read exon ratio
Average intron size
h
ScISOr-Seq (ONT)
Per-read exon ratio
Average intron size
i
No. unique features
Internal exon pairs
TSS-internal exon pairs
PolyA-internal exon pairs
Genes
Subsampling fraction

Supplementary Figure 3: Technical performance of SnISOr-Seq. a. *Left*: Chart shows normalized sequencing coverage of SnISOr-Seq (blue) along the length of annotated transcripts, compared with our previously published method ScISOr-Seq (red), Illumina 10x 3' single-nuclei RNA-seq (black) and Illumina paired-end bulk sequencing (purple).*Right*: Normalized sequencing coverage of 76bp in-silico fragmented SnISOr-Seq long reads (red) compared to Illumina 10x 3' single-nuclei RNA-seq (black) X-axis indicates normalized position from 5' to 3'. **b.** Box plots show the read length of sequenced reads for ScISOr-Seq (red) compared to SnISOr-Seq (green) (n=657703,4426614 reads). **c.** Box plots show the exon count of sequenced reads for ScISOr-Seq and SnISOr-Seq (ONT and PacBio) (n=657703,4426614,2908266,2941553 reads). **d.** Histogram of distance to the nearest CAGE peak for reads considered 5' complete. **e-f.** Bar plots show the fraction of reads that are complete at the 5'-end (e) and 3'-end (f) of the molecule. Genes were split into three categories based on their average intron length: <1 kb, 1-10 kb, and >10 kb. **g-h**. Bar plots showing, for SnISOr-Seq (g) and ScISOr-Seq (h) the per-read exon count divided by the average exon count for annotated transcripts in that gene. As above, genes were split into three categories based on their average intron length: <1 kb (blue), 1-10 kb (red), and >10 kb (green). Values on the Y-axis can go above 1 when more than the average annotated exons are sequenced in a sample (n=602268,3133706,690640,233788,378103,45812 reads). **i.** Saturation plot showing the number of unique internal exon pairs (black), TSS-internal exon pairs (blue), polyA-internal exon pairs (black) and genes (orange), across various subsampled sequencing depths. For box plots: center line, median; box limits, upper and lower quartiles; whiskers, 1.5x interquartile range.

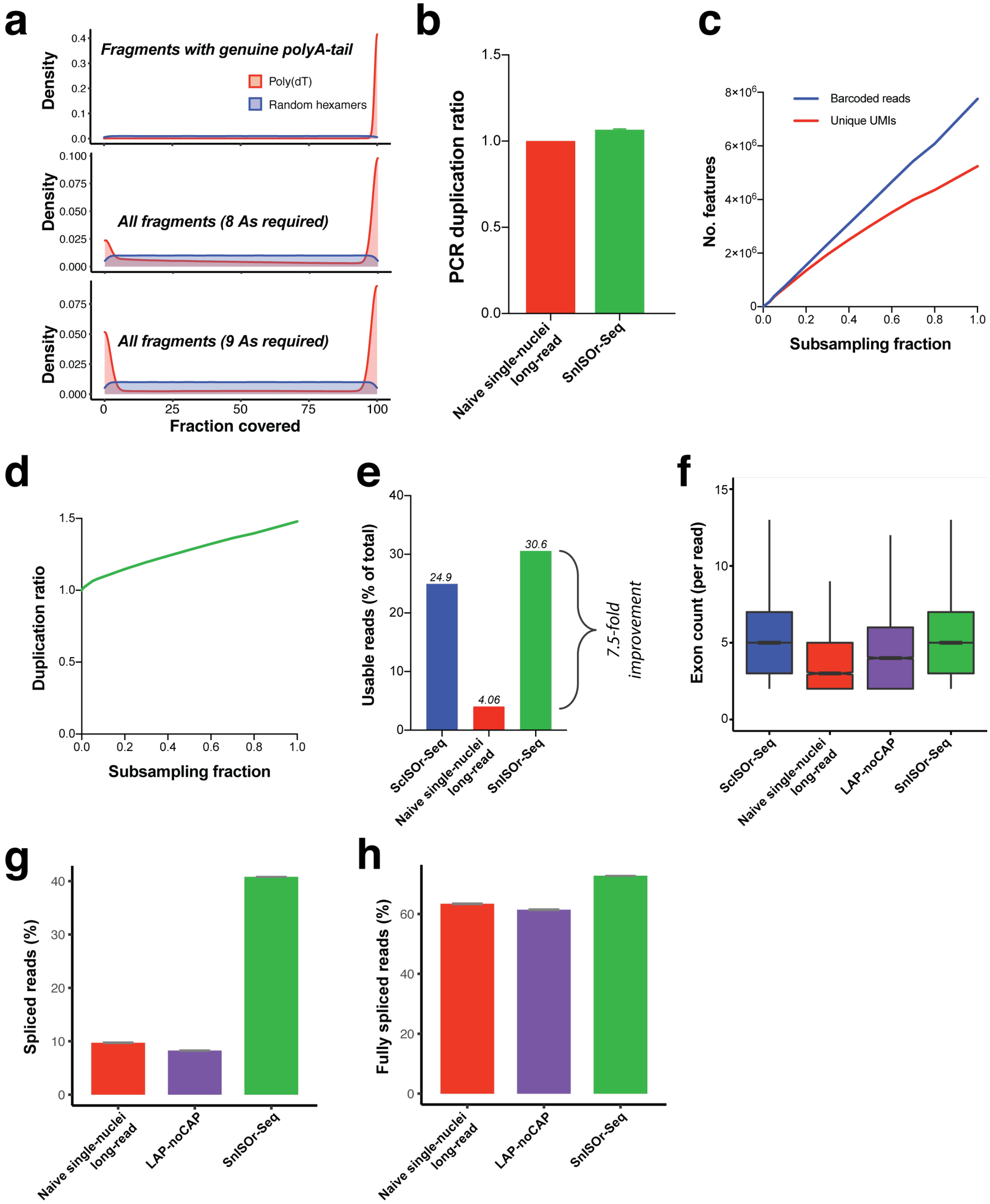

Supplementary Figure 4: Further technical performance of SnISOr-Seq. a. Density plot of fraction of annotated transcript covered when primed with oligo(dT) or random hexamers. Molecules with polyA tail (top), all molecules with poly(dT) priming for 8 (middle) and 9 (bottom) A's in a 10 bp window. X-axis indicates percent of fragment captured after priming simulation. **b.** Bar plots show the PCR duplication ratio (number of barcoded reads divided by number of UMIs). SnISOr-Seq library has been subsampled (n=390,420 barcoded reads; 366,379 unique UMIs) to the same

depth as the naïve single-nuclei library (n=222,920 barcoded reads; 222,642 unique UMIs). **c.** Plot shows the number of barcoded reads (blue) and UMIs (red) for SnISOr-Seq across various subsampled sequencing depths. **d.** Plot shows the PCR duplication ratio for SnISOr-Seq across various subsampled sequencing depths. **e.** Bar plots show the fraction of reads that are usable (i.e., barcoded and on-target) **f.** Box plots show the exon count per read. For box plots: center line, median; box limits, upper and lower quartiles; whiskers, 1.5x interquartile range. **g.** Bar plots (n= 226157,678632,8827449) show the percent of barcoded reads that are spliced **h.** Bar plots (n=118617,138512,8132265) show the percent of fully spliced reads with no intron retention. **b,e-h**: ScISOr-Seq (blue), naïve single-nuclei long-read RNA sequencing (red), LAP-noCAP (purple), SnISOr-Seq (green). **b,g-h** Error bars represent SE of the point estimate.

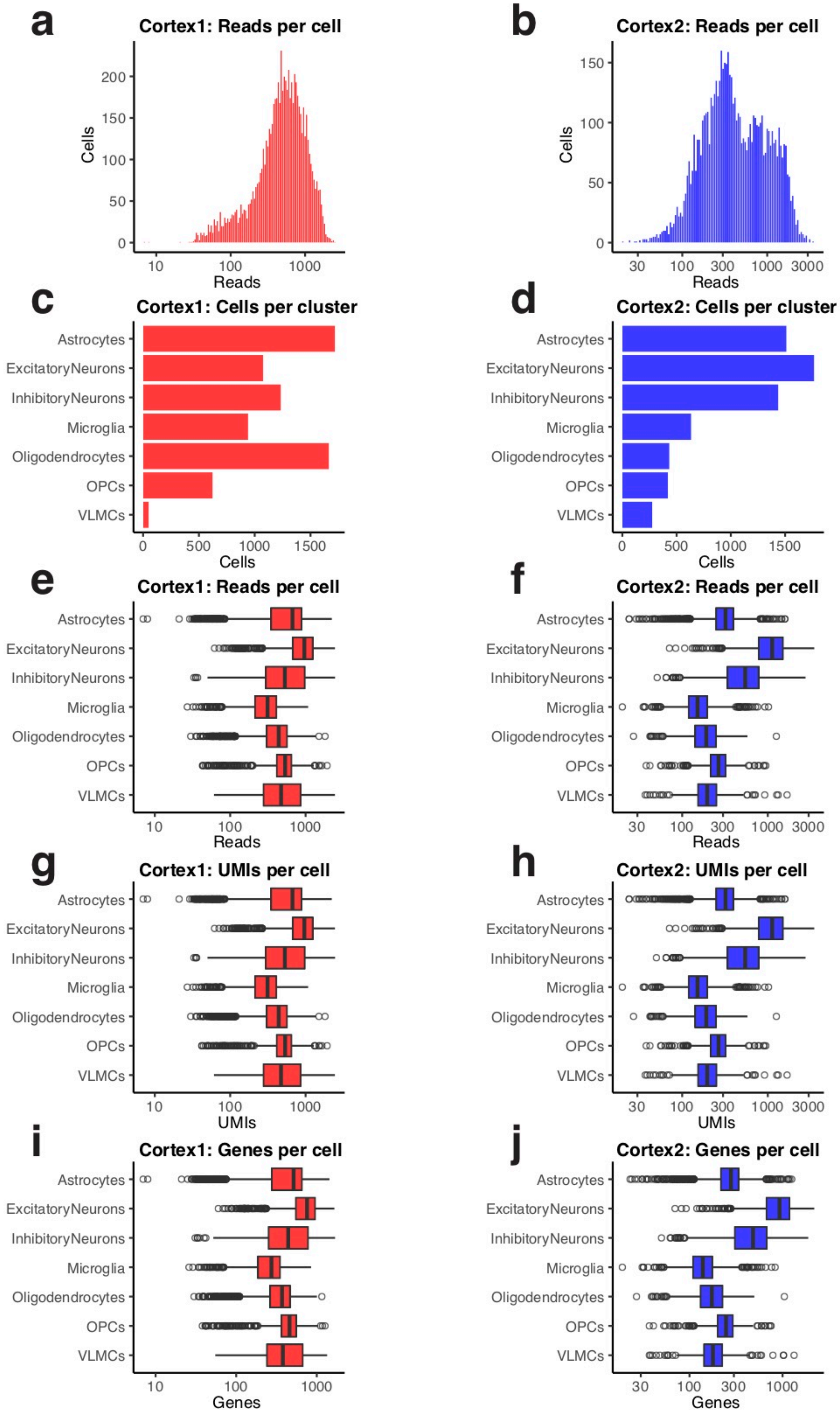
a
Cortex1: Reads per cell
Cells
Reads
b
Cortex2: Reads per cell
Cells
Reads
c
Cortex1: Cells per cluster
d
Cortex2: Cells per cluster
Astrocytes
ExcitatoryNeurons
InhibitoryNeurons
Microglia
Oligodendrocytes
OPCs
VLMCs
Cells
e
Cortex1: Reads per cell
f
Cortex2: Reads per cell
g
Cortex1: UMIs per cell
UMIs
h
Cortex2: UMIs per cell
i
Cortex1: Genes per cell
Genes
j
Cortex2: Genes per cell

Supplementary Figure 5: Long-read sequencing statistics. a-b. Histogram of reads per single nucleus with reads on the X-axis and number of single nuclei sequenced on the Y-axis. Cortex1 on the left (red) and Cortex2 on the right (blue). **c-d.** Barplot of the number of single-nuclei recovered per cell type. Color of bar indicates sample, i.e. Cortex1 on the left and Cortex2 on the right. **e-f.** Boxplots of reads per single nucleus, grouped by cell type. **g-h.** Boxplots of UMIs per single nucleus, grouped by cell type. **i-j.** Boxplots of genes per single nucleus, grouped by cell type, with Cortex1 on the left and Cortex2 on the right. For box plots: center line, median; box limits, upper and lower quartiles; whiskers, 1.5x interquartile range; outliers shown as circles.

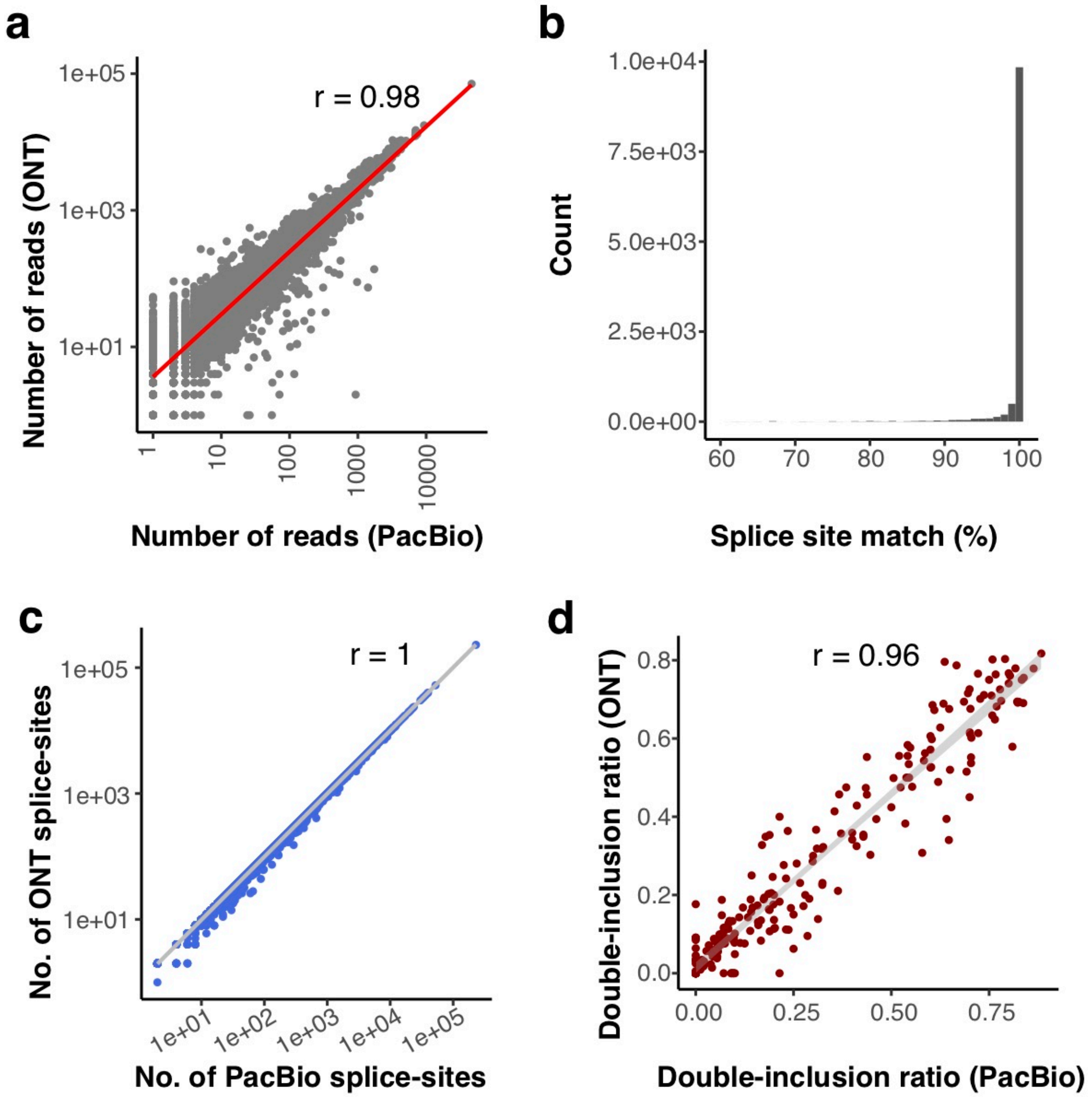

Supplementary Figure 6: Comparison of ONT and PacBio long-read sequencing libraries. a. Scatter plot of the number of reads per gene from both technologies. X and Y axis are presented on a log10 scale **b.** Histogram of splice sites as a percentage of PacBio sites that were also found in ONT **c.** Scatter plot of number of splice sites per gene in PacBio (X-axis) vs. ONT (Y-axis) for identical molecules **d.** Scatter plot of double inclusion levels for exon pairs with sufficient coverage i.e., 10 reads per exon pair. **a, c-d.** Pearson's r indicated. **b-d** Statistics calculated for identical molecules i.e., molecules sharing the same gene, barcode, and UMI.

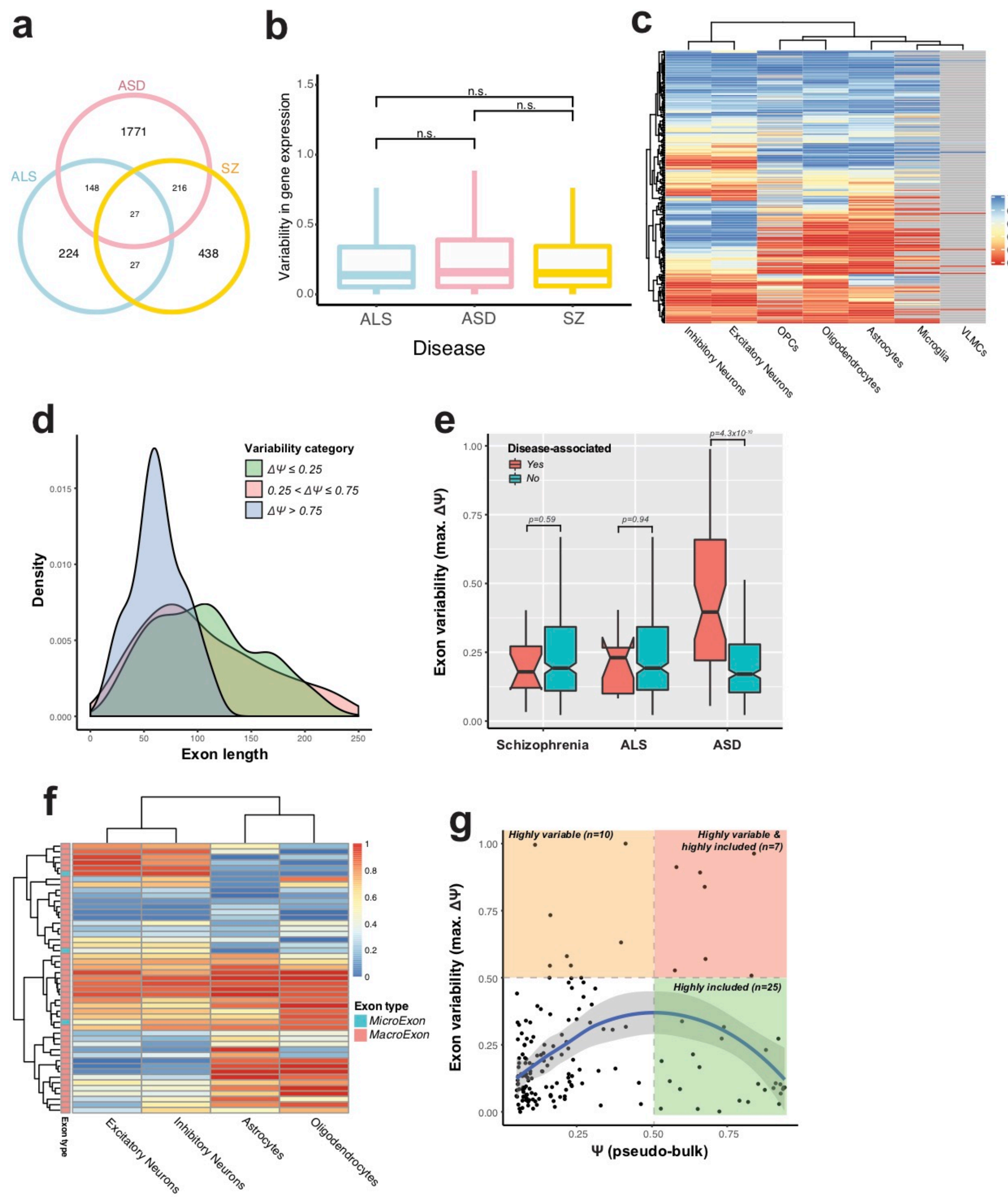

Supplementary Figure 7: Disease association and replicable observations for single exon usage. a. Venn diagram showing the number of genes from which disease-associated exons are derived. **b**. Box plots denoting variability in gene expression, calculated as the difference between the maximum and minimum values of log normalized TPMs across cell types. p-values calculated using two-sided Wilcoxon rank sum test. **c.** Heatmap of exon variability in Cortex1 considering other broad cell types. **d-g.** Panels correspond to Fig. 3b-e, but using data from Cortex2. **g.** Regression curve with 95% confidence interval obtained using the loess fit. ASD: Autism Spectrum Disorder; ALS: Amyotrophic Lateral Sclerosis; SZ: Schizophrenia. For box plots: center line, median; box limits, upper and lower quartiles; whiskers, 1.5x interquartile range.

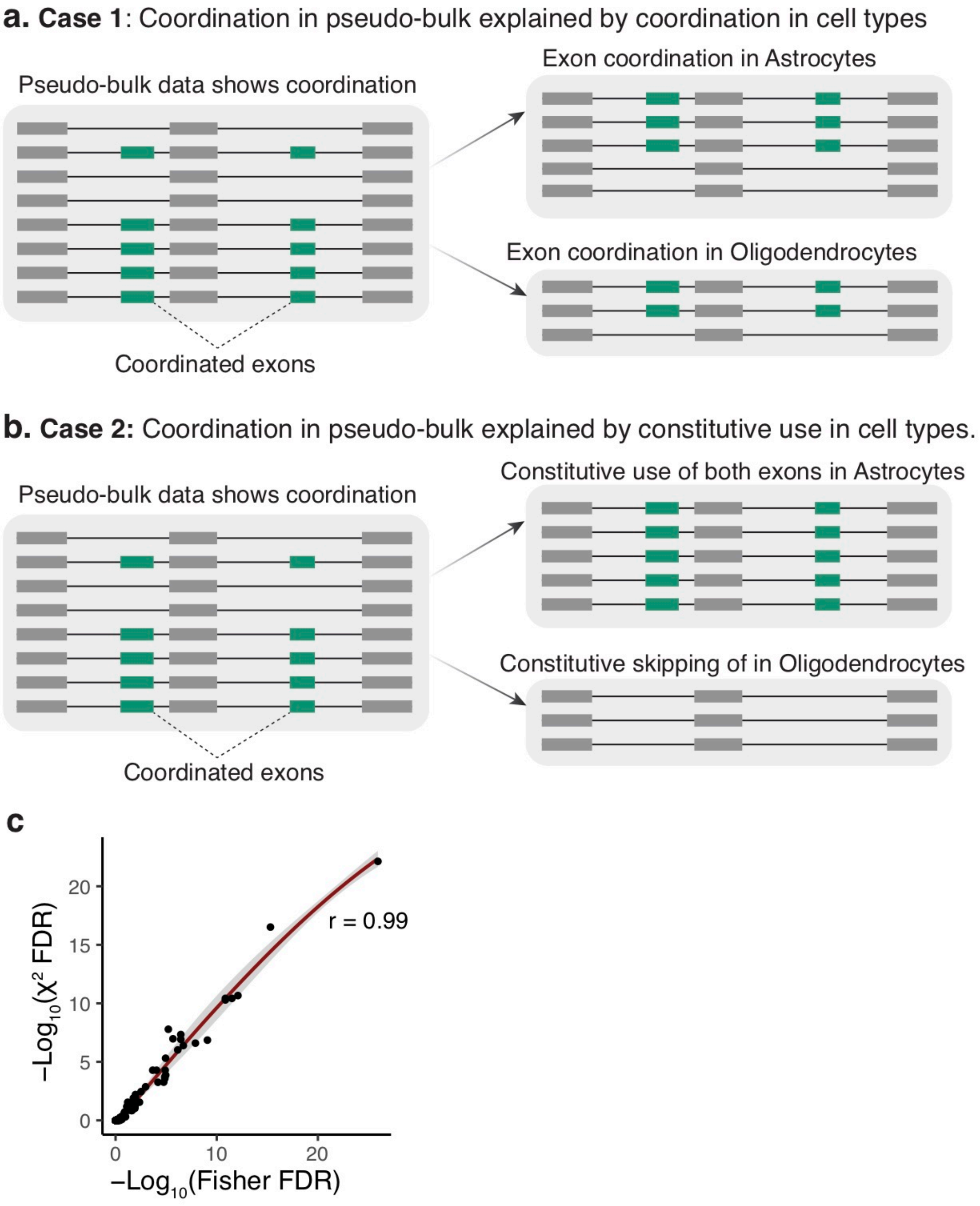

Supplementary Figure 8: Investigation of pseudo-bulk exon coordination in cell-types. Coordination in pseudo-bulk explained by **a.** coordination in cell types **b.** constitutive use in cell types. **a-b.** Alternative coordinated exons indicated in green **c.** Scatter plot of the -$\log_{10}$ BY corrected p-value using the χ^2 test versus the -$\log_{10}$ BH corrected p-value obtained using two-sided Fisher's exact test. Regression lines with 95% confidence interval obtained using the loess fit.

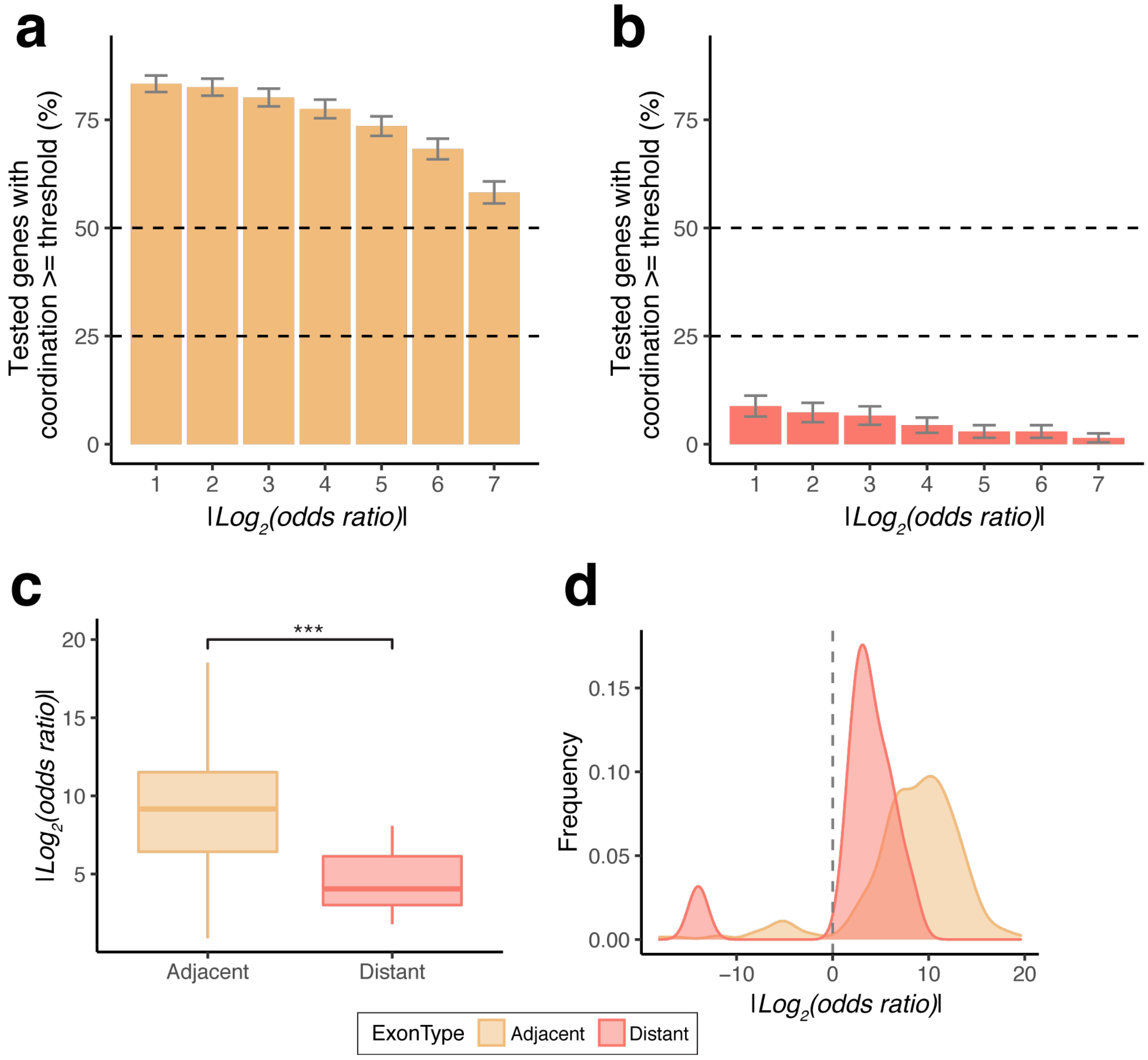

Supplementary Figure 9: Replicable coordination of adjacent and distant exon pairs. a-b Bar plots for Cortex2 showing percent of tested genes in pseudo-bulk with significant exon coordination for adjacent (a; n=378) and distant (b; n=136) exon pairs like in Fig 4b-c. Error bars indicate SE of the point estimate **c.** Box plots of the |log-odds-ratio| for significant genes plotted against adjacent (n=316) and distant (n=9) exon pairs on the X-axis. p-value obtained from two-sided Wilcoxon rank sum test. For box plots: center line, median; box limits, upper and lower quartiles; whiskers, 1.5x interquartile range. **d.** Density plot for the log-odds-ratio for adjacent and distant exon pairs from a-b. Significance=*P<0.05; **P<0.005; ***P<0.001; n.s.=non-significant.

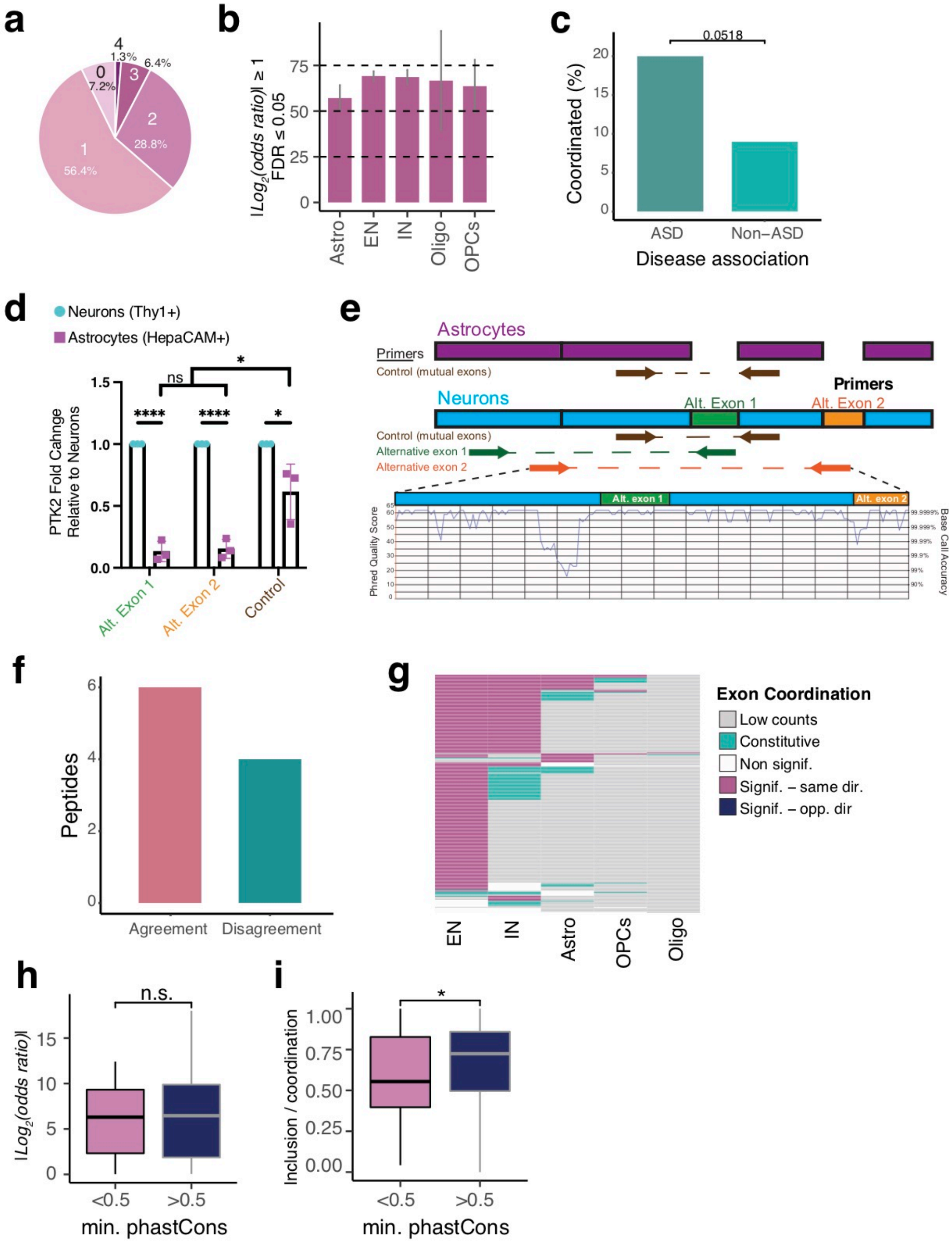

Supplementary Figure 10: Exon coordination patterns are replicable and orthogonally validated. a. Corresponds to Fig. 5a but using data from Cortex2. **b.** Bar plot of percentage of tested exon-pairs from Cortex2 that were significantly coordinated. Cell type on the X-axis (n=49,305,137,3,11). **c.** Bar plot showing percent of distant coordinated exon pairs split by ASD-association (n=40,200). p-value obtained from two-sided Fisher's exact test. **d-e.** qRT-PCR fold change across isolated neurons and astrocytes for Alt. Exons 1-2 and control exons. mutually shared between neurons and astrocytes. Expression was compared using the comparative CT method and data is shown normalized to neuronal expression. Statistical analyses performed using two-sided unpaired t-test (n=3 biological replicates (GW 19-20)). Error bars represent SD of the mean. **f.** Bar plot showing the number of peptides derived from ASD-associated exons that had agreement and

disagreement with the cell-type specific exon variability. **g-i.** Panels correspond to Fig. 5g-i, but using data from Cortex2. **h-i**. n=49, 240 exon pairs respectively for min. phastCons <0.5 and >0.5. P-values obtained from two-sided Wilcoxon rank sum test. EN: Excitatory neurons, IN: Inhibitory Neurons, Astro: Astrocytes, Oligo: Oligodendrocytes, OPCs: Oligodendrocyte Precursor Cells. For box plots: center line, median; box limits, upper and lower quartiles; whiskers, 1.5x interquartile range. **b,e**: Error bars indicate SE of the point estimate. Significance=*P<0.05; ****P < 0.0001; ns= not significant.

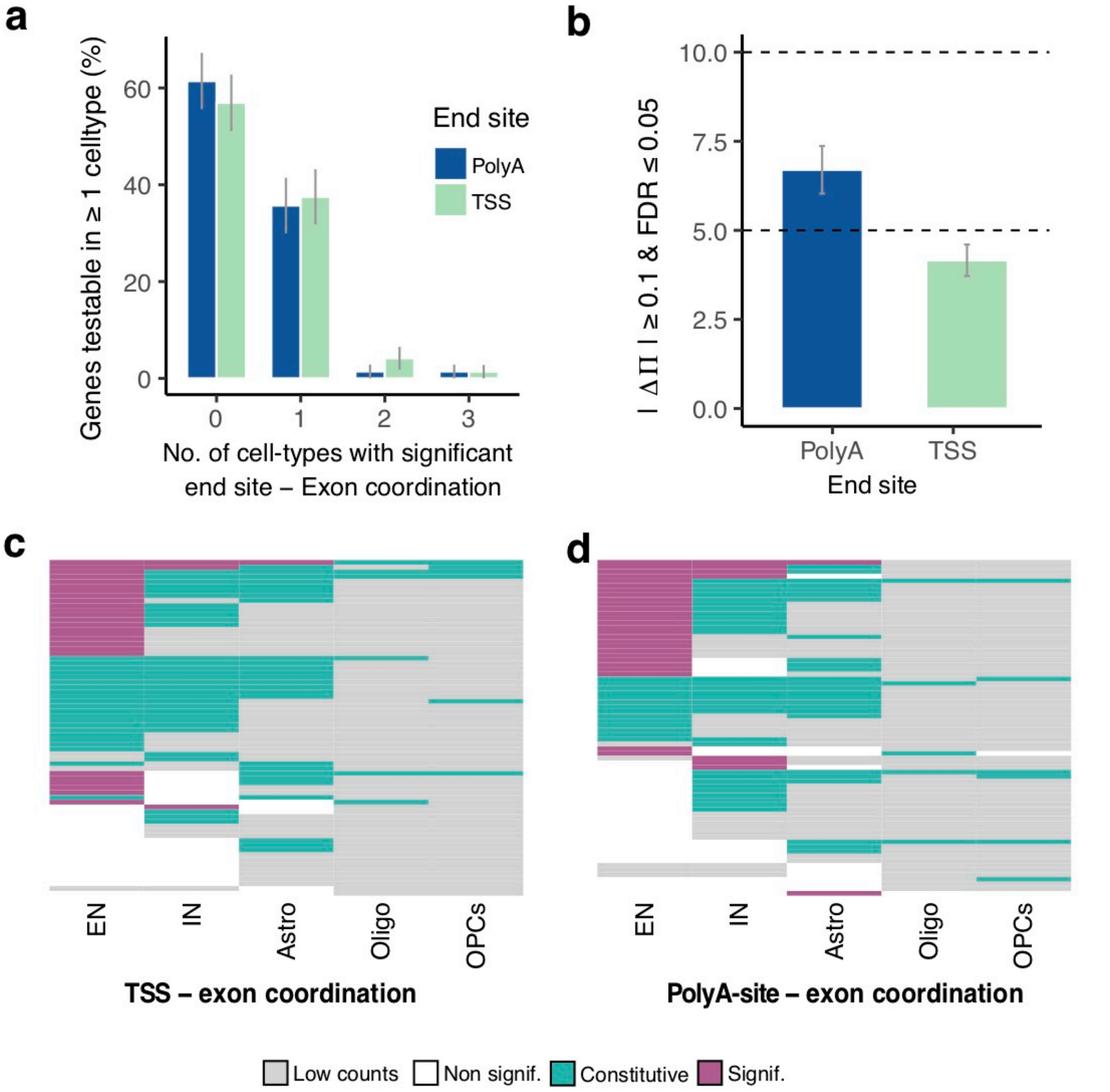

Supplementary Figure 11: Cell type mediated exon - end site coordination in Cortex2. a. Bar chart of number of cell types where an exon-end site pair is significant given significance in pseudo-bulk (n=70 TSS, n=72 PolyA-sites). **b.** Bar chart of percent of tested genes in pseudo-bulk with significant exon-end site coordination on the Y-axis and end site on X-axis. Color of bar (a-b) indicates whether the end site associated with an exon is a TSS (n=2043, blue) or polyA-site (n=1403, green). Error bars (a-b) indicate SE of the point estimate. **c-d.** Panels correspond to Fig. 6c,g but using data from Cortex2 (n=70 TSS-exon pairs: c, n=72 PolyA-site-exon pairs: d). EN: Excitatory neurons, IN: Inhibitory Neurons, Astro: Astrocytes, Oligo: Oligodendrocytes, OPCs: Oligodendrocyte Precursor Cells.

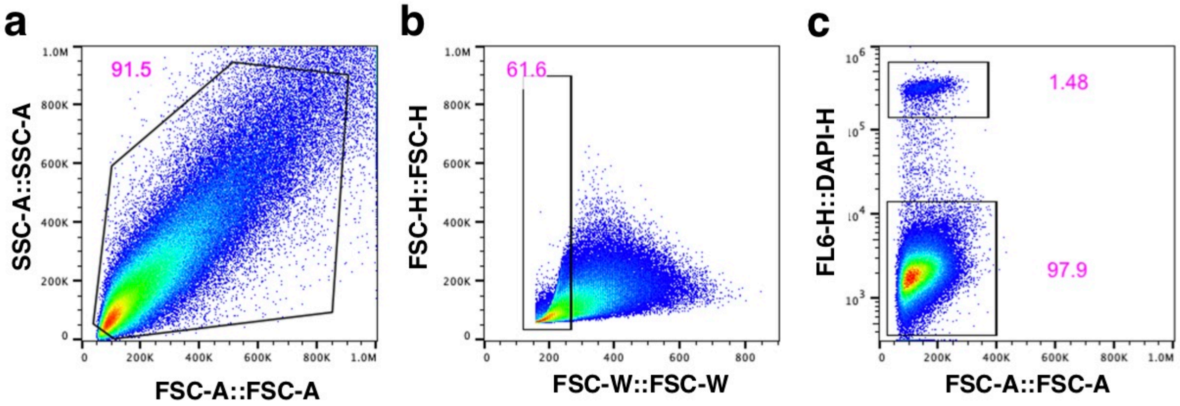

Supplementary Figure 12: FACS gating strategy. Representative image of FACS gating strategy used. **a**. Most events were included in the FSC/SSC gate. **b.** Singlets were identified from FSC-W/FSC-H gate. **c.** Lastly, a distinct DAPI+ population was sorted.

Supplementary Table 1: Long-read sequencing statistics

Sample	Platform	Flow cell	No. reads	Mapped	On-target rate (%)	Avg. read length (bp)
Cortex1 (LAP-CAP)	ONT	FCtx1_ONT_Run1.fastq.gz	18,668,000	15,309,783	72.33	1,284
		FCtx1_ONT_Run2.fastq.gz	65,880,057	54,592,985	71.93	1,158
		FCtx1_ONT_Run3.fastq.gz	71,328,400	59,578,396	69.27	1,123
Cortex2 (LAP-CAP)	ONT	FCtx2_ONT_Run1.fastq.gz	44,079,800	38,466,551	68.81	864
		FCtx2_ONT_Run2.fastq.gz	61,576,667	52,461,421	74.63	965
Cortex1 (LAP-CAP)	PacBio	FCtx1_PacBio_Run1.fastq.gz	1,994,964	1,736,962	77.52	1,112
		FCtx1_PacBio_Run2.fastq.gz	2,913,665	2,536,474	77.45	1,112
		FCtx1_PacBio_Run3.fastq.gz	2,559,272	2,237,625	78.04	1,140
		FCtx1_PacBio_Run4.fastq.gz	3,219,346	2,803,921	77.59	1,114
		FCtx1_PacBio_Run5.fastq.gz	2,447,659	2,125,993	77.54	1,105
		FCtx1_PacBio_Run6.fastq.gz	2,334,405	2,025,196	77.46	1,108
		FCtx1_PacBio_Run7.fastq.gz	2,731,126	2,402,928	78.57	1,149
		FCtx1_PacBio_Run8.fastq.gz	2,013,024	1,766,104	78.37	1,151
Cortex2 (LAP-CAP)	PacBio	FCtx2_PacBio_Run1.fastq.gz	1,069,301	919,510	60.63	1,099
		FCtx2_PacBio_Run2.fastq.gz	297,808	256,278	61.00	1,083
		FCtx2_PacBio_Run3.fastq.gz	549,338	471,551	61.06	1,069
		FCtx2_PacBio_Run4.fastq.gz	402,627	346,060	61.00	1,067

		FCtx2_PacBio_Run5.fastq.gz	1,557,832	1,335,563	61.56	1,077
		FCtx2_PacBio_Run6.fastq.gz	754,952	647,741	61.49	1,088
		FCtx2_PacBio_Run7.fastq.gz	194,487	163,288	77.01	935
Cortex1 (naïve)	PacBio	FCtx1_PacBio_preCAP.fastq.gz	1,121,497	933,889	23.49	1,219
Cortex2 (naïve)	PacBio	FCtx2_PacBio_preCAP.fastq.gz	1,487,748	1,264,707	24.74	1,233
Cortex1 (LAP-noCAP)	PacBio	FCtx1_PacBio_LAP_noCAP.fastq.gz	1,811,710	1,570,167	20.07	1,247

ONT: Oxford Nanopore Technologies; PacBio: Pacific Biosciences; LAP: linear/asymmetric PCR; CAP: capture-based target enrichment.
