# Supplementary material for: Single-nuclei isoform RNA sequencing unlocks barcoded exon connectivity in frozen brain tissue
Source: Nat Biotechnol. 2022 Mar 7;40(7):1082–92. doi: 10.1038/s41587-022-01231-3 (PMC9287170; doi:10.1038/s41587-022-01231-3)
Supplement: Supplementary file 2 — Reporting Summary [file 41587_2022_1231_MOESM2_ESM.pdf]

nature research

Corresponding author(s): Hagen U. Tilgner

Last updated by author(s): Jan 14, 2022

Reporting Summary

Nature Research wishes to improve the reproducibility of the work that we publish. This form provides structure for consistency and transparency in reporting. For further information on Nature Research policies, see our Editorial Policies and the Editorial Policy Checklist.

Statistics

For all statistical analyses, confirm that the following items are present in the figure legend, table legend, main text, or Methods section.

n/a	Confirmed	
☐	☒	The exact sample size (*n*) for each experimental group/condition, given as a discrete number and unit of measurement
☐	☒	A statement on whether measurements were taken from distinct samples or whether the same sample was measured repeatedly
☐	☒	The statistical test(s) used AND whether they are one- or two-sided<br>*Only common tests should be described solely by name; describe more complex techniques in the Methods section.*
☐	☒	A description of all covariates tested
☐	☒	A description of any assumptions or corrections, such as tests of normality and adjustment for multiple comparisons
☐	☒	A full description of the statistical parameters including central tendency (e.g. means) or other basic estimates (e.g. regression coefficient) AND variation (e.g. standard deviation) or associated estimates of uncertainty (e.g. confidence intervals)
☐	☒	For null hypothesis testing, the test statistic (e.g. *F*, *t*, *r*) with confidence intervals, effect sizes, degrees of freedom and *P* value noted<br>*Give P values as exact values whenever suitable.*
☒	☐	For Bayesian analysis, information on the choice of priors and Markov chain Monte Carlo settings
☐	☒	For hierarchical and complex designs, identification of the appropriate level for tests and full reporting of outcomes
☐	☒	Estimates of effect sizes (e.g. Cohen's *d*, Pearson's *r*), indicating how they were calculated

Our web collection on statistics for biologists contains articles on many of the points above.

Software and code

Policy information about availability of computer code

Data collection	Cells were sorted using the FlowJo (v10) software. RNA-seq reads were base-called using SMRT Link (PacBio reads) or Guppy (ONT reads). Reads were then mapped to the human genome using STARlong (v2.5.2b, PacBio reads) or minimap2 (v2.17-r943-dirty, ONT reads).
Data analysis	Barcodes were identified using cellranger (v3.1.0, short-reads) or custom code (see below). Seurat (v3.1.5) was used for single-cell processing and visualization, cell clustering was done with the Louvain algorithm within Seurat. Pre-processing for long-read analysis was done using the scisorseqr package (v0.1.2). The source code generated for this paper is publicly available at https://github.com/noush-joglekar/sn-code.

For manuscripts utilizing custom algorithms or software that are central to the research but not yet described in published literature, software must be made available to editors and reviewers. We strongly encourage code deposition in a community repository (e.g. GitHub). See the Nature Research guidelines for submitting code & software for further information.

Data

Policy information about availability of data

All manuscripts must include a data availability statement. This statement should provide the following information, where applicable:

- Accession codes, unique identifiers, or web links for publicly available datasets
- A list of figures that have associated raw data
- A description of any restrictions on data availability

All data used for this study is publicly available on GEO under the accession token GSE178175. All data supporting the findings of this study are provided within the paper and its supplementary information. Source data for the main figures can be found at https://github.com/noush-joglekar/sn-code

Field-specific reporting

Please select the one below that is the best fit for your research. If you are not sure, read the appropriate sections before making your selection.

☒ Life sciences ☐ Behavioural & social sciences ☐ Ecological, evolutionary & environmental sciences

For a reference copy of the document with all sections, see nature.com/documents/nr-reporting-summary-flat.pdf

Life sciences study design

All studies must disclose on these points even when the disclosure is negative.

Sample size	No sample size calculation was performed. We only used a sample size of 2 as human brain samples are difficult to source.
Data exclusions	Nuclei were excluded from downstream analysis if they had exceptionally low (<200) or high (>7500) RNA counts or had high (>4%) fraction of reads mapping to mitochondrial genes.
Replication	Replication was performed n=2 times. All key findings observed in Cortex1 were replicated in Cortex2
Randomization	Not relevant to our study as we only used two human brain samples and no treatment was applied to them.
Blinding	Not relevant to our study as we only used two human brain samples and no treatment was applied to them.

Reporting for specific materials, systems and methods

We require information from authors about some types of materials, experimental systems and methods used in many studies. Here, indicate whether each material, system or method listed is relevant to your study. If you are not sure if a list item applies to your research, read the appropriate section before selecting a response.

Materials & experimental systems

n/a	Involved in the study	
☒	☐	Antibodies
☒	☐	Eukaryotic cell lines
☒	☐	Palaeontology and archaeology
☒	☐	Animals and other organisms
☐	☒	Human research participants
☒	☐	Clinical data
☒	☐	Dual use research of concern

Methods

n/a	Involved in the study	
☒	☐	ChIP-seq
☐	☒	Flow cytometry
☒	☐	MRI-based neuroimaging

Human research participants

Policy information about studies involving human research participants

Population characteristics	All human tissues we use were supplied to us as de-identified post-mortem samples and are thus considered "non-human subjects research". This is in compliance with NIH, Weill Cornell and Emory University policies, protocols, and guidance in working with human tissues
Recruitment	-
Ethics oversight	Regional and Institutional Committee of Science and Research Ethics of Scientific Council of Health,Code of Ethics of the World Medical Association,Semmelweis University Regional Committee of Science and Research Ethics, Stanford University

Note that full information on the approval of the study protocol must also be provided in the manuscript.

Flow Cytometry

Plots

Confirm that:

☒ The axis labels state the marker and fluorochrome used (e.g. CD4-FITC).

☒ The axis scales are clearly visible. Include numbers along axes only for bottom left plot of group (a 'group' is an analysis of identical markers).

☒ All plots are contour plots with outliers or pseudocolor plots.

☒ A numerical value for number of cells or percentage (with statistics) is provided.

Methodology

Sample preparation	Approximately 30 mg of frozen tissue of each sample was dissected in a sterile dish on dry ice and transferred to a 2 mL glass tube containing 1.5 mL nuclei pure lysis buffer (MilliporeSigma, catalog no. L9286) on ice. Tissue was completely minced and homogenized to nuclei suspension by sample grinding with Dounce homogenizers. The nuclei suspension was filtered by loading through a 35 μm diameter filter and followed by centrifuging 5 min at 600 g and 4℃. The nuclei pellet was collected and washed with cold wash buffer, which consisted of the following reagents: 1X PBS , 20 mM DTT, 1%BSA , 0.2U/μl RNase inhibitor for three times. After removing the supernatant from the last wash, the nuclei were resuspended in 1 mL of 0.5 ug/ml DAPI and the concentration was estimated by using Countess II cell counter.
Instrument	Sony MA900 (Sony Biotechnology), Countess II cell counter (Thermo Fisher Scientific, catalog no. A27977).
Software	FlowJo V10.
Cell population abundance	Among the post-sort populations include over 100K nuclei, around 80%-90% are identified as the aiming nuclei population, which are determined by the DAPI-positive rate readout from Countess II cell counter (Thermo Fisher Scientific, catalog no. A27977).
Gating strategy	Most events were included in the FSC/SSC gate. Singlets were identified from FSC-W/FSC-H gate. Lastly, a distinct DAPI+ population was sorted.

☒ Tick this box to confirm that a figure exemplifying the gating strategy is provided in the Supplementary Information.
